# Altered DNA base excision repair profile in brain tissue and blood in Alzheimer’s disease

**DOI:** 10.1186/s13041-016-0237-z

**Published:** 2016-05-28

**Authors:** Meryl S. Lillenes, Alberto Rabano, Mari Støen, Tahira Riaz, Dorna Misaghian, Linda Møllersen, Ying Esbensen, Clara-Cecilie Günther, Per Selnes, Vidar T. V. Stenset, Tormod Fladby, Tone Tønjum

**Affiliations:** Department of Microbiology, Oslo University Hospital, Oslo, Norway; Department of Microbiology, University of Oslo, Oslo, Norway; Fundación Centro Investigación Enfermedades Neurológicas (CIEN), Madrid, Spain; Department of Clinical Molecular Biology and Laboratory Sciences (EpiGen), Division of Medicine, Akershus University Hospital and University of Oslo, Lørenskog, Norway; Norwegian Computing Center, Oslo, Norway; Department of Neurology, Faculty Division, Akershus University Hospital, University of Oslo, Lørenskog, Norway; Department of Microbiology, University of Oslo, Oslo University Hospital, Postbox 4950 Nydalen, Oslo, NO-0424 Norway

**Keywords:** Alzheimer’s disease, DNA repair, Base excision repair, DNA glycosylase OGG1, PARP1, APE1, DNA polymerase Polβ, Brain tissue

## Abstract

**Background:**

Alzheimer’s disease (AD) is a progressive, multifactorial neurodegenerative disorder that is the main cause of dementia globally. AD is associated with increased oxidative stress, resulting from imbalance in production and clearance of reactive oxygen species (ROS). ROS can damage DNA and other macromolecules, leading to genome instability and disrupted cellular functions. Base excision repair (BER) plays a major role in repairing oxidative DNA lesions. Here, we compared the expression of BER components APE1, OGG1, PARP1 and Polβ in blood and postmortem brain tissue from patients with AD, mild cognitive impairment (MCI) and healthy controls (HC).

**Results:**

BER mRNA levels were correlated to clinical signs and cerebrospinal fluid biomarkers for AD. Notably, the expression of BER genes was higher in brain tissue than in blood samples. *Polβ* mRNA and protein levels were significantly higher in the cerebellum than in the other brain regions, more so in AD patients than in HC. Blood mRNA levels of *OGG1* was low and *PARP1* high in MCI and AD.

**Conclusions:**

These findings reflect the oxidative stress-generating energy-consumption in the brain and the importance of BER in repairing these damage events. The data suggest that alteration in BER gene expression is an event preceding AD. The results link DNA repair in brain and blood to the etiology of AD at the molecular level and can potentially serve in establishing novel biomarkers, particularly in the AD prodromal phase.

**Electronic supplementary material:**

The online version of this article (doi:10.1186/s13041-016-0237-z) contains supplementary material, which is available to authorized users.

## Background

Alzheimer's disease (AD) is a progressive, multifactorial neurodegenerative disease affecting 24 million individuals worldwide, with an incidence expected to double within 2030 [1]. AD is thus by far the most important contributor to cognitive decline and dementia globally. AD is characterized by impairment in memory and cognition, synaptic dysfunction, neuronal loss, extracellular amyloid beta (Aβ) plaques and intracellular neurofibrillary tangles (NFTs) composed of fibrillar aggregates of hyperphosphorylated tau in the brain [2]. Low Aβ-42 in cerebrospinal fluid (CSF) may reflect deposition of amyloid in brain plaques, while high total tau (T-tau) and phosphorylated tau (P-tau) in CSF may reflect neuronal degeneration [3, 4]. These CSF biomarkers are used in the clinic for diagnosis of patients with AD [2, 5, 6]. However, it remains a challenge to diagnose early stages of multifactorial sporadic (non-familial) AD. This is consistent with the hypothesis of multiple underlying factors leading to sporadic AD, whose etiology remains poorly understood.

Age is the principal risk factor for AD and ageing is associated with cumulative oxidative stress. A leading hypothesis proposes that high levels of oxidized nucleic acids in brain cells can lead to neuronal dysfunction in patients with AD [7–9], and the evidence linking oxidative damage to neurodegeneration is overwhelming [10–12]. Consistent with this, reactive oxygen species (ROS) can damage macromolecules such as lipids, proteins, DNA and RNA. At the same time, it is clear that genome dynamics and defects in DNA repair processes under the oxidative stress induced in neurodegeneration hold a key to main understanding in neuropathogenesis. Because environmental genotoxic components generally fail to pass through the blood–brain barrier, it is thought that endogenous ROS are the primary cause of oxidative DNA damage inside neurons and glial cells [13, 14].

DNA damage is balanced with repair in a homeostatic process, and imbalance occurs when the damage exceeds repair, causing cellular senescence, genome mutation or apoptosis. These features are more abundant in old cells than in young cells [15, 16]. A high level of DNA damage can be particularly deleterious in post-mitotic cells as they do not self-renew through cell proliferation. However, oxidative damage to DNA and RNA may be a cause or consequence of neurodegeneration, reflecting either increased production of ROS or reduced DNA repair [14]. Previous studies show that the amount and the capacity to repair DNA damage varies with age and varies in different brain regions, and that pathological features of AD are higher in brain regions where more DNA damage is detected [14, 17, 18]. BER is the major pathway for repair of oxidative DNA damage [19] and epidemiology studies have associated reduced BER capacity with neurodegenerative diseases such as AD [20, 21]. The first step in BER usually involves excision of a damaged base by a lesion-specific DNA glycosylase, e.g. when OGG1 removes 8oxoG from an 8oxoG:C base pair [22], which generates an abasic (apurinic/apyrimidinic; AP) site [23]. In the next step in BER, AP-endonuclease 1 (APE1) hydrolytically cleaves the phosphodiester backbone at the AP site, after which polymerase beta (Polβ) performs end-processing and gap-filling DNA synthesis, and finally, DNA ligase seals the nick [19]. Polβ is the major DNA polymerase and tissue-specific expression levels of Polβ has been reported [24]. Poly (ADP-ribose) polymerase (PARP1) plays a role in many DNA repair reactions, including BER [25–27].

In AD, brain pathology can be observed many years before cognitive decline is clinically evident. CSF biomarkers indicate brain pathology compatible with mild cognitive impairment (MCI) preceding AD [6], and central nervous system amyloid deposition can be determined either by CSF Aβ42 or amyloid PET scans in preclinical AD [28]. At the same time, AD brain pathology may be present in the absence of cognitive impairment. However, inexpensive, non-invasive biomarkers for pre-dementia are lacking, and additional biomarkers to identify individuals at risk for dementia and other types of cognitive disease/decline are non-existent.

We have previously shown that there are alterations in mRNA levels of *APE1* and *OGG1* in tissue from different brain regions of the tg-ArcSwe mouse model and that these occurred prior to the development of AD pathology [29]. As mouse models only represent models of imposed AD pathology and do not reflect human AD in complexity, we asked if BER mRNA levels were altered between human AD patients and healthy controls (HC) in blood and in brain tissue. The present study explores the relationships between the quantitative analysis of mRNA transcripts for the BER genes encoding APE1, Polβ, OGG1 and PARP1 and CSF biomarkers of AD and clinical signs of cognitive decline in blood from a clinical cohort (*n* = 166) as well as in a second cohort of postmortem samples of different brain parts (hippocampus, cerebellum, entorhinal cortex and frontal cortex) from AD patients (*n* =42) and HC (*n* = 9). The results show that alterations in the BER gene expression profile in blood is an early event evident already at the prodromal stage of AD. This could be a useful indicator for disease progression/initiation status in patients with preclinical AD.

## Methods

### Participants and clinical assessment

This cross-sectional study was performed using two separate cohorts. The first cohort included 166 live individuals at different disease stages relevant to AD: 41 AD patients with dementia, 28 patients with MCI due to AD pathology, 45 patients with MCI and 24 patients with subjective cognitive impairment (SCI) and 28 HC recruited at the Memory Clinic at Akershus University Hospital Additional file 1: Table S1). Assessments of these patients included lumbar puncture (LP) for CSF sample, blood draw, brain MRIs (except in cases where CT scan was preferred over MRI), formal cognitive testing including mini mental status evaluation (MMSE) [30], Cognistat [31], geriatric depression testing and neurological examination. For further information on diagnostic eligibility criteria and patient groups, see the Additional file 1.

The second cohort included freshly frozen postmortem human brain samples of the hippocampus, entorhinal cortex, frontal cortex and cerebellum obtained from 42 histopathologically confirmed AD patients (average age 82.4, age range 57–98 years, 20 males/22 females) and 9 human controls displaying no AD histopathology at time of death (average age 61.7, age range 46–84 years, 5 males/4 females, for more patient characteristics see Additional file 1: Table S2). The brain samples were provided by Fundación Centro Investigación Enfermedades Neurológicas (CIEN)/Carlos III Health Research Institute (ISCIII).

### CSF and blood analysis

CSF and blood analysis were performed in cohort one. Blood samples were drawn by venipuncture and CSF samples (5 mL) were drawn by LP under spinal anesthesia or during an otherwise scheduled diagnostic procedures. LP was performed between 09–12 AM. The concentration of T-tau, P-tau and Aβ-42 in CSF was measured using protocols developed at Akershus University Hospital (Additional file 1: Tables S3 and S4). APOE was determined using DNA isolated from blood samples, and the APOE allele frequencies are listed in Additional file 1: Table S5.

### RNA isolation from blood and brain samples and quantitative real-time (RT) qPCR

Blood samples were collected in PAXgene RNA collection tubes (PreAnalytiX GmbH, Switzerland). The samples were stored at −80 °C until use. Total RNA was extracted using the PAXgene Blood RNA kit (PreAnalytiX GmbH, Switzerland) in accordance with the manufacturer’s recommendations. Human brain samples were weighed and homogenized using MagNA Lyser Green Beads (Roche Diognostics GmbH, Mannheim, Germany) and lysis buffer from PureLink® RNA Mini Kit (Ambion, Texas, USA) in a MagNA Lyser instrument (Roche Diognostics GmbH, Mannheim, Germany). Total RNA was isolated using PureLink® RNA Mini Kit according to manufacturer’s recommendations. The RNA concentration of both blood and brain tissue was determined using an ND-1000 spectrophotometer (NanoDrop technologies, Saveen & Werner AB, Sweden); RNA purity, integrity and yield were confirmed using Agilent 2001 Bioanalyzer and RNA 6000 Nano Kit (Agilent technologies, California, USA) according to the recommendations of the manufacturer. RNA samples with unsatisfactory purity (blood RIN < 7, brain RIN < 5) were isolated again until satisfactory purity and RIN was obtained or dismissed. cDNA was prepared from 1000 ng total RNA from blood and brain tissue in 100 μL using the High cDNA reverse transcription kit (Applied Biosystems) according to the recommendations from the manufacturer (Invitrogen, US).

### Quantitative real-time (RT) qPCR of RNA from brain samples

mRNA transcripts encoding *OGG1, APE1, Polβ* and *PARP1* were quantified by real time q-PCR (qRT-PCR) using methodology and equipment by Applied Biosystems (Foster City, CA, USA). Complete TaqMan gene expression assay information is listed in Additional file 1: Table S6 and relative mRNA levels (mean, range) for all groups are listed in Additional file 1: Table S7. To control for differences in efficiency of the reverse transcription and real-time PCR reactions and pipetting errors, normalization to a reference gene was also included (in addition to other normalization procedures, please see Additional file 1 for further information). The selection of the reference gene was performed after a validation of 32 candidate reference genes using the TaqMan Human Endogenous Control Plates (Applied Biosystems, Foster City, CA, USA) according to the recommendations of the manufacturer. Glyceraldehyde phosphate dehydrogenase (GAPDH) was identified as one of the most stable reference genes with the additional preferred Ct value (<25) and was thus chosen as the endogenous reference gene. qRT-PCR was performed by using the StepOnePlus™ system in 96 well plates with TaqMan Gene expression assays according to the recommendations of the manufacturer. Samples were held at 95 °C for 10 min, cycling was at 95 °C for 15 min and 58 °C for 1 hour for 55 cycles. The melting curve cycle was at 95 °C for 15 min, 60 °C for 1 hour and 95 °C for 15 min. All plates were set up according to the relative standard curve method with separate standard curves on each plates for both target and reference gene. All standard curves were made from the same sample, thus also functioning as a positive control and reference sample between runs. The relative gene expression of target genes was calculated using the relative standard curve method; briefly, this is the difference in Ct value and y-intercept between the target gene and a calibrator sample, divided by the slope and normalized to the reference gene (GAPDH) and adjusted for minute efficiency (see Additional file 1 for more analysis details). Deep sequencing of cDNA from a subset of brain samples was performed in parallel using the Illumina HiSeq-2000 sequencer (50 base pairs single reads, mean read depth 22379938,5 (SD 167932,5), BGI, China). Fragments per kilobase Million (FPKM)) of APE1, OGG1, Polβ and PARP1 values of the individual samples are listed in Additional file 1: Table S8.

### Proteomics analyses by high-end mass spectrometry

Peptide characterization and quantitation were performed by electrospray-based high resolution mass spectrometry (Q-Exactive, Thermo-Fischer). Brain tissue was lysed in lysis solvent containing 2%SDS/10 mM Tris–HCl, pH7.5 supplemented with protease inhibitor cocktail (EDTA free, Roche) and PhosStop (Roche) and disrupted with MagNa Lyser instrument (Roche) in cycles until fully lysed. Prior to trypsin digestion, 100 μg of protein lysates were precipitated over night with acetone at −20 °C. Air dried protein pellet was re-suspended in 10 μl of 8 M urea. Proteins were reduced with 1 μl of 10 mM DTT (Sigma-Aldrich) followed by alkylation with 1 μl 50 mM iodoacetamide (Sigma-Aldrich). The samples were then diluted with 50 mM ammonium bicarbonate and digested with 1:100 of trypsin (sequencing grade modified, Promega, USA). The digested samples were fractionated by anion exchange column and samples were run on a Q-Exactive (Thermo Scientific, Germany) mass spectrometer coupled directly to an nLC (EASY 1000, Thermo Scientific, Germany) using a data-dependent Top10 method. Mass spectrometry results were searched using MaxQuant software against the human UniProt database with proteome ID:UP000005640. For further methodological specifications, please see the Supplementary material (Additional file 1: Table S9.).

### Statistical analyses

To test for differential expression of BER genes among AD and non-AD individuals in the brain samples, a linear mixed model was fitted to each gene in each brain region. The fixed effects were disease status (AD or HC), brain region (hippocampus, cerebellum, frontal cortex or entorhinal cortex) and the interaction between disease status and brain region. A random individual effect was included to account for the potential correlation between measurements in different regions within the same brain. The significance of the overall interaction was assessed by an F-test. If the overall interaction was not significant, the effect of disease status was assessed directly with a t-test (Table 1). If the interaction effect was significant, each brain region was analyzed separately by fitting a reduced model. It included the fixed effect of brain region, interaction between disease status and brain region, and random effects. For each brain region, a t-test was used to test whether the interaction of disease status and brain region was significantly different from zero (Table 2). A significance level of 0.05 was used for this analysis. For each clinical covariate (diagnosis and CSF biomarkers) and each gene, a linear regression model was fitted with the gene expression as the dependent variable. APOE was adjusted for by including it in the model together with the clinical covariate. If the clinical covariate was categorical, each level of the covariate was compared to a reference level. The significance of the clinical covariates was assessed with a Wald test (Table 3). A two-sample t-test was used to test for significant differences in gene expression between brain and blood and between different brain parts (Table 4) Due to the large number of tests in the last mentioned three analysis (Tables 2, 3 and 4), the results in these tables should be interpreted with care. In this setting, it is not clear how one should correct for multiple testing, as many of the tests are dependent. Applying e.g. the Bonferroni criterion would be too strict. However, many of the results were significant even when correcting the p-values with the Bonferroni correction, and are thus reliable. The Pearson correlation was calculated between the RNA deep sequencing data and the RT-qPCR gene expression data.

**Table 1:** *APE1, OGG1, Polβ* and *PARP1* mRNA levels in brain regions of AD patients and healthy controls

Overall interaction between diagnosis and brain region			Specific interactions between diagnosis and brain region		
Base excision repair enzyme	*p*-value	*p*-value	Brain region	Effect	*p*-value
APE1	0.034	- *	→		
			Frontal cortex	0.13	0.61
			Cerebellum	0.17	0.49
			Hippocampus	0.33	0.22
			Entorhinal cortex	−0.62	0.016 *
Polβ	0.0026	- *	→		
			Frontal cortex	0.40	0.85
			Cerebellum	9.93	4.6E-06 ****
			Hippocampus	0.24	0.92
			Entorhinal cortex	1.12	0.63
PARP1	2.40 E-09	- *	→		
			Frontal cortex	0.33	0.63
			Cerebellum	−2.43	0.00042***
			Hippocampus	0.16	0.004 **
			Entorhinal cortex	2.71	0.00018***
OGG1	0.25	0.57			

The significance of the specific interaction effects of diagnosis and brain region are only tested if the overall interaction effect is significant, otherwise the significance of the main effect of diagnosis is tested directly. *p-values*: * < 0.05, ** < 0.01, *** < 0.001, **** < 0.0001

**Table 2:** Comparison of mRNA levels of *APE1, OGG1, Polβ* and *PARP1* in different brain regions of AD patients (AD) and healthy controls (HC)

Base excision repair enzyme	Reference brain part	Compared brain part	AD *p*-value	HC *p*-value
APE1	Frontal cortex	Cerebellum	0.88	0.2
	Frontal cortex	Hippocampus	0.23	0.8
	Frontal cortex	Entorhinal cortex	0.8	0.3
	Cerebellum	Hippocampus	0.27	0.58
	Cerebellum	Entorhinal cortex	0.68	0.77
	Hippocampus	Entorhinal cortex	0.18	0.68
OGG1	Frontal cortex	Cerebellum	0.11	0.55
	Frontal cortex	Hippocampus	0.66	0.83
	Frontal cortex	Entorhinal cortex	0.6	0.67
	Cerebellum	Hippocampus	0.4	0.6
	Cerebellum	Entorhinal cortex	0.31	0.87
	Hippocampus	Entorhinal cortex	0.99	0.77
Polβ	Frontal cortex	Cerebellum	0.002 **	0.005**
	Frontal cortex	Hippocampus	0.34	0.21
	Frontal cortex	Entorhinal cortex	0.23	0.54
	Cerebellum	Hippocampus	0.00005****	0.01**
	Cerebellum	Entorhinal cortex	0.0002****	0.005**
	Hippocampus	Entorhinal cortex	0.78	0.17
PARP1	Frontal cortex	Cerebellum	0.77	0.64
	Frontal cortex	Hippocampus	0.96	0.44
	Frontal cortex	Entorhinal cortex	0.66	0.43
	Cerebellum	Hippocampus	0.84	0.38
	Cerebellum	Entorhinal cortex	0.47	0.37
	Hippocampus	Entorhinal cortex	0.67	0.92

*p-values*: * < 0.05, ** < 0.01, *** < 0.001, **** < 0.0001

**Table 3:** Association of clinical diagnosis, CSF biomarkers and age with mRNA levels of*APE1, OGG1, Polβ* and *PARP1* in blood

Clinical determinant Diagnosis	Base Excision Repair Enzyme	Association recorded	*p-value*
AD dementia	APE1	0.04	0.598
MCI/AD		−0.06	0.508
MCI		−0.06	0.425
SCI		0.05	0.570
AD dementia	OGG1	−0.13	0.0324 *
MCI/AD		−0.13	0.0410 *
MCI		−0.15	0.0083 **
SCI		−0.08	0.1941
AD dementia	Polβ	0.22	0.0395 *
MCI/AD		0.12	0.3165
MCI		0.03	0.7295
SCI		0.11	0.3235
AD dementia	PARP1	0.27	0.018 *
MCI/AD		0.46	0.00014 ***
MCI		0.33	0.00150 **
SCI		0.48	0.00007 ****
CSF biomarkers			
No CSF pathology	APE1	−0.05	0.492
Tau pathology		0.09	0.320
Aβ pathology		−0.08	0.453
Tau and Aβ pathology		0.02	0.856
No CSF pathology	OGG1	−0.12	0.0164 *
Tau pathology		−0.17	0.0103 *
Aβ pathology		−0.14	0.0468 *
Tau and Aβ pathology		−0.09	0.1826
No CSF pathology	Polβ	0.02	0.8508
Tau pathology		0.25	0.0352 *
Aβ pathology		0.14	0.2794
Tau and Aβ pathology		0.19	0.1180
No CSF pathology	PARP1	0.43	0.00002 ****
Tau pathology		0.40	0.0013 **
Aβ pathology		0.38	0.0053 **
Tau and Aβ pathology		0.32	0.0089 **
Age			
Change over time: average effect per year	APE1	−0.004	0.14
	OGG1	−0.0006	0.768
	PARP1	−0.009	0.0196 *
	Polβ	0.010	0.00599 **

The parameters are compared to healthy controls. *p-values*: * < 0.05, ** < 0.01, *** < 0.001, **** < 0.0001

**Table 4:** mRNA levels of *APE1, OGG1, Polβ* and *PARP1* in brain parts compared with blood

Gene	Brain parts	Mean NGE All brain parts	Mean NGE Blood	*p*-value
AD patients				
APE1	All brain parts	1.73	1.06	2.69E-14 *
OGG1	All brain parts	4.38	0.90	<2.20e-16 *
Polβ	All brain parts	8.31	0.99	1.42E-10 *
PARP1	All brain parts	3.45	1.14	<2.20E-16 *
APE1	Frontal cortex	1.55	1.06	1.0E-04 *
	Cerebellum	2.19	1.06	7.76E-10 *
	Hippocampus	1.47	1.06	3.65E-05 *
	Entorhinal cortex	1.73	1.06	8.41E-08 *
OGG1	Frontal cortex	3.23	0.90	2.2E-11 *
	Cerebellum	6.51	0.90	1.16E-11 *
	Hippocampus	3.91	0.90	5.44E-11 *
	Entorhinal cortex	3.93	0.90	9.62E-14 *
Polβ	Frontal cortex	1.75	0.99	1.30E-04 *
	Cerebellum	28.62	0.99	8.34E-16 *
	Hippocampus	1.58	0.99	8.40E-05 *
	Entorhinal cortex	2.37	0.99	1.30E-10 *
PARP1	Frontal cortex	2.17	1.14	4.67E-08 *
	Cerebellum	2.86	1.14	3.12E-10 *
	Hippocampus	3.87	1.14	9.28E-11 *
	Entorhinal cortex	4.87	1.14	1.54E-11 *
Healthy controls				
APE1	All brain parts	1.76	1.02	5.40E-05 *
OGG1	All brain parts	3.27	1.02	1.20E-05 *
Polβ	All brain parts	6.20	0.78	2.47E-03
PARP1	All brain parts	2.85	0.87	2.10E-04 *
APE1	Frontal cortex	1.41	1.02	2.10E-02
	Cerebellum	2.03	1.02	1.70E-04 *
	Hippocampus	1.16	1.02	1.40E-01
	Entorhinal cortex	2.36	1.02	3.70E-02
OGG1	Frontal cortex	2.69	1.02	3.70E-02
	Cerebellum	5.18	1.02	2.90E-04 *
	Hippocampus	1.85	1.02	5.70E-02
	Entorhinal cortex	3.01	1.02	1.30E-01
Polβ	Frontal cortex	1.36	0.78	3.40E-02
	Cerebellum	18.69	0.78	4.70E-04 *
	Hippocampus	1.36	0.78	2.80E-03
	Entorhinal cortex	1.20	0.78	2.30E-02
PARP1	Frontal cortex	1.83	0.87	4.60E-02
	Cerebellum	5.31	0.87	1.30E-02
	Hippocampus	1.81	0.87	5.50E-03
	Entorhinal cortex	2.15	0.87	1.20E-02

Abbreviations: *NGE* normalized mRNA levels, *significant p-values*: *

## Results

### mRNA levels of DNA repair enzymes are higher in brain tissue than in blood samples

**Fig. 1:**
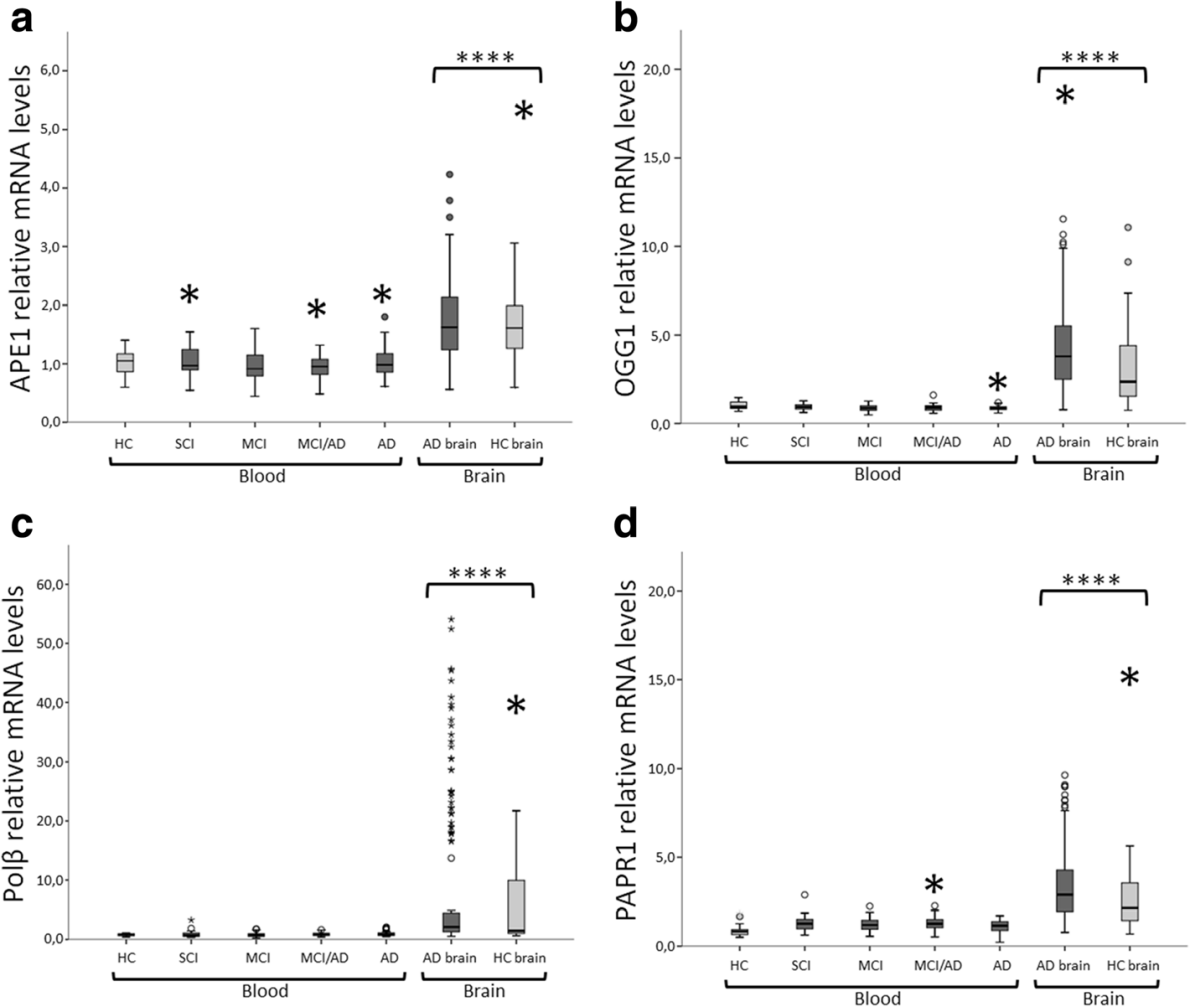
mRNA levels of *APE1*, *OGG1*, *Polβ* and *PARP1* in blood compared to human brain tissue (mean ± 2 SEM), *p-values*: * < 0.05, ** < 0.01, *** < 0.001, **** < 0.0001. **a** Relative mRNA levels of *APE1* in blood compared to levels in human brain tissue. **b** Relative mRNA levels of *OGG1* in blood compared to levels in human brain tissue. **c** Relative mRNA levels of *Polβ* in blood compared to levels in human brain tissue. **d** Relative mRNA levels of *PARP1* in blood compared to levels in human brain tissue. The box plots shows that mRNA levels of all enzymes are significantly higher (*p <0.0001*) in brain tissue compared to blood, both in healthy controls and AD patients. Abbreviations: AD = Alzheimer’s disease, SCI = patients with subjective cognitive impairment, MCI = patients with mild cognitive impairment, MCI/AD = patients with mild cognitive impairment due to AD pathology

In order to investigate the relationship between BER mRNA levels in blood and brain tissue, we compared the gene expression from the different brain regions with gene expression in blood in AD patients and HC. The mRNA levels for *APE1, OGG1, Polβ* and *PARP1* was in general significantly higher in brain tissue compared to blood (Fig 1, Table 4). The difference was greater in AD patients for all four BER components in general, showing significant differences also when comparing blood to each brain region for all components and all brain regions under study (Table 4). There was also a significant difference between mRNA levels in blood and brain tissue in HC for *APE1, OGG1* and *PARP1* in general. When comparing blood to each brain region, significant differences were found for *APE1, OGG1* and *Polβ* in cerebellum only (Table 4). These findings are also evidence of the intact integrity of the postmortem brain tissue mRNA and high BER transcriptional level compared to blood.

### Altered mRNA levels of *APE1*, *Polβ* and *PARP1* in tissue from various brain regions

**Fig. 2:**
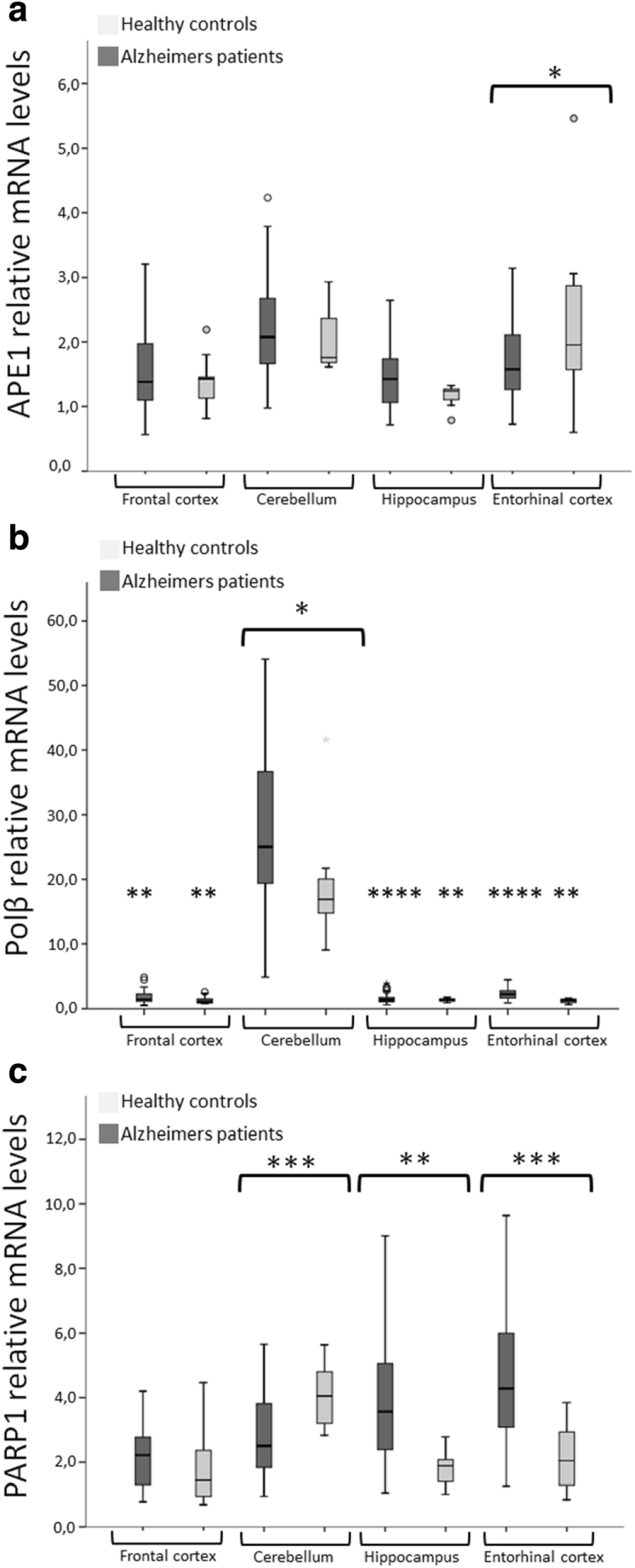
*APE1* and *Polβ* mRNA levels in brain parts from AD patients and healthy controls (HC) (mean ± 2 SEM)**,** *p-values*: * < 0.05, ** < 0.01, *** < 0.001, **** < 0.0001. **a** The box plot shows that mRNA levels of *APE1* are significantly lower (*p <0.05*) in entorhinal cortex of AD patients compared to HC. **b** The box plot shows that mRNA levels of *Polβ* are significantly higher (*p <0.05*) in cerebellum of AD patients compared to HC. *Polβ* mRNA is also significantly higher in the cerebellum compared to all other brain parts in both HC and AD patients. **c** The box plot shows that *PARP1* mRNA levels were significantly lower in AD cerebellum (*p < 0.0005*) compared to HC cerebellum and significantly higher in AD hippocampus (*p < 0.005*) and entorhinal cortex (*p < 0.0001*) compared to the same regions in HC. *OGG1* mRNA levels did not differ between AD and HC brain regions

Quantitative real time PCR was used to assess mRNA levels in tissue from four different brain regions from 42 AD patients and 9 HC. *APE1* mRNA was significantly lower in the entorhinal cortex of AD patients (*p <0.05*) than in the same cortical area in healthy controls (Fig. 2a, Table 1). *Polβ* mRNA was significantly higher in the cerebellum of AD patients (*p <0.00001*) than in the same brain region in healthy controls (Fig. 2b, Table 1) and *PARP1* mRNA was significantly lower in AD cerebellum (*p < 0.0005*) compared to HC cerebellum and significantly higher in AD hippocampus (*p < 0.005*) and entorhinal cortex (*p < 0.0001*) compared to the same regions in HC (Fig. 2c, Table 1). *OGG1* mRNA levels did not differ between AD and HC brain regions.

*Polβ* mRNA was higher in cerebellum compared to all other brain regions in both AD and HC. Particularly, *Polβ* mRNA was significantly different between cerebellum and hippocampus (*p < 0.00005*) and entorhinal cortex (*p <0.001*) and frontal cortex (*p <0.01*) within AD patients and between cerebellum and hippocampus (*p <0.01*), entorhinal cortex (*p <0.005*) and frontal cortex (*p <0.005*) within HC (Fig. 2b, Table 2). Expression of *OGG1* and *PARP1* did not differ between AD patients and HC in general or in any specific brain region.

### Altered *OGG1* and *PARP1* mRNA levels in the prodromal phases of AD

**Fig. 3:**
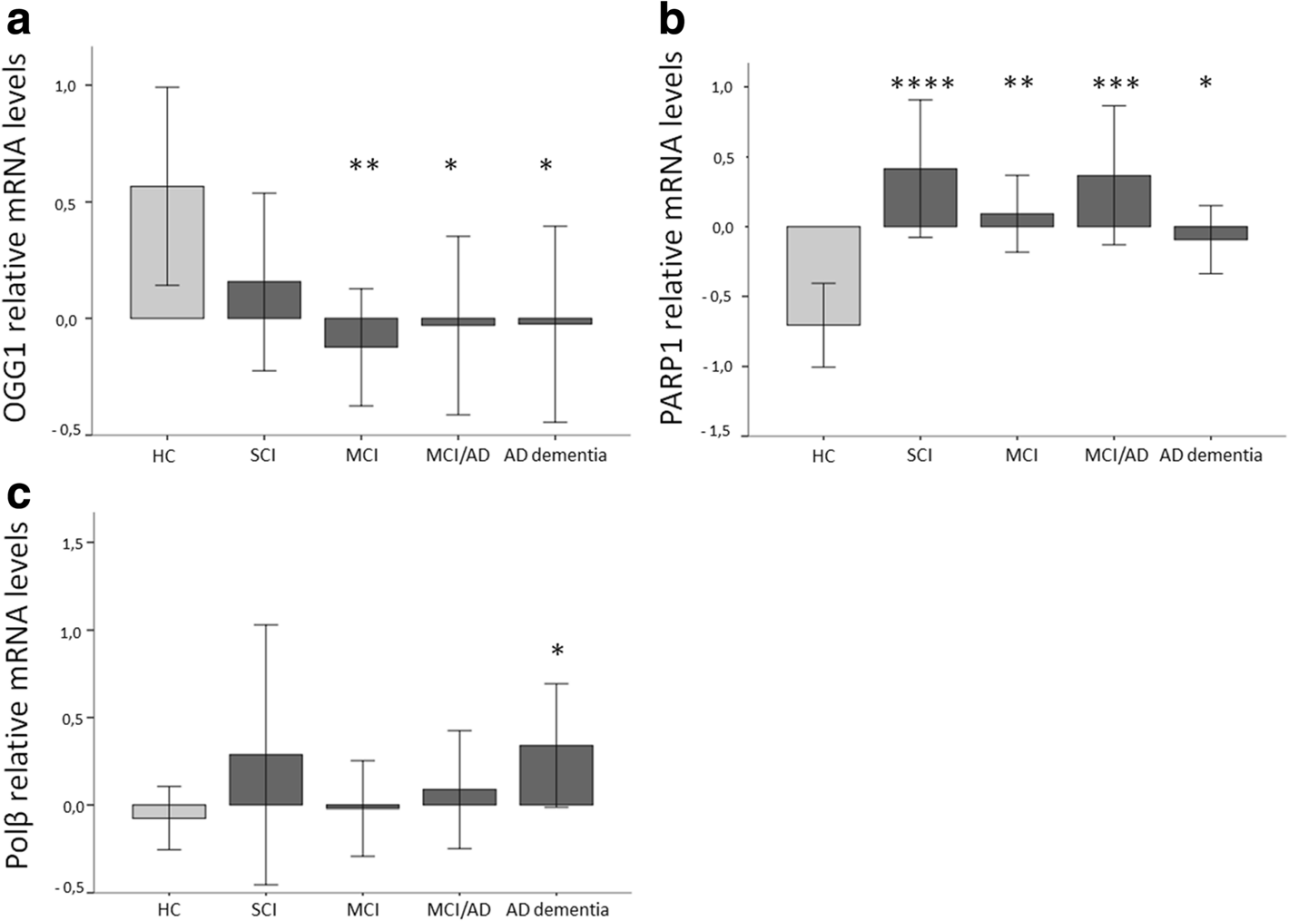
Relative mRNA levels of *OGG1, PARP1* and *Polβ* in selected diagnostic groups (mean ± 2 SEM) in AD patients with dementia, mild cognitive impairment (MCI) due to AD (MCI/AD), MCI, subjective cognitive impairment (SCI) and healthy controls (HC). *p-values*: * < 0.05, ** < 0.01, *** < 0.001, **** < 0.0001. **a** The box plot show that the mRNA levels of *OGG1* are significantly lower in the diagnosis groups: MCI (*p <0.01*), MCI/AD (*p <0.05*) and AD dementia (*p <0.05*) compared to HC. **b** The box plot show that the mRNA levels of *PARP1* are significantly higher in the diagnosis groups: SCI (p <0.0001), MCI (*p <0.01*), MCI/AD (*p <0.001*) and AD dementia (*p <0.05*) compared to HC. **c** The box plot show that the mRNA levels of *Polβ* are significantly higher in the diagnosis group AD dementia (*p <0.05*) compared to HC

The progression in the BER repair mRNA profile in the different phases of AD progression was investigated. We compared the gene expression of *APE1, OGG1, Polβ* and *PARP1* in 41 AD patient with dementia, 28 patients with MCI due to AD pathology, 45 patients with MCI and 24 patients with SCI and 28 HC. *OGG1* mRNA was lower in AD dementia (*p <0.05*), MCI/AD (*p <0.05*) and patients with MCI (*p <0.01*) than in HC (Fig 3a and Table 3). *PARP1* mRNA was higher in patients with AD dementia (*p <0.05*), MCI/AD (*p <0.01*), MCI (*p <0.01*), and SCI (*p <0.01*) than in HC (Fig. 3b and Table 3). *Polβ* mRNA was higher in patients with AD dementia (*p <0.05*) compared to the other groups (Fig. 3c, Table 3). The abundance of the *APE1* mRNA levels did not correlate significantly with any clinical diagnostic marker in any cohort subgroup.

### Correlation of OGG1 and PARP1 mRNA levels with CSF biomarkers

**Fig. 4:**
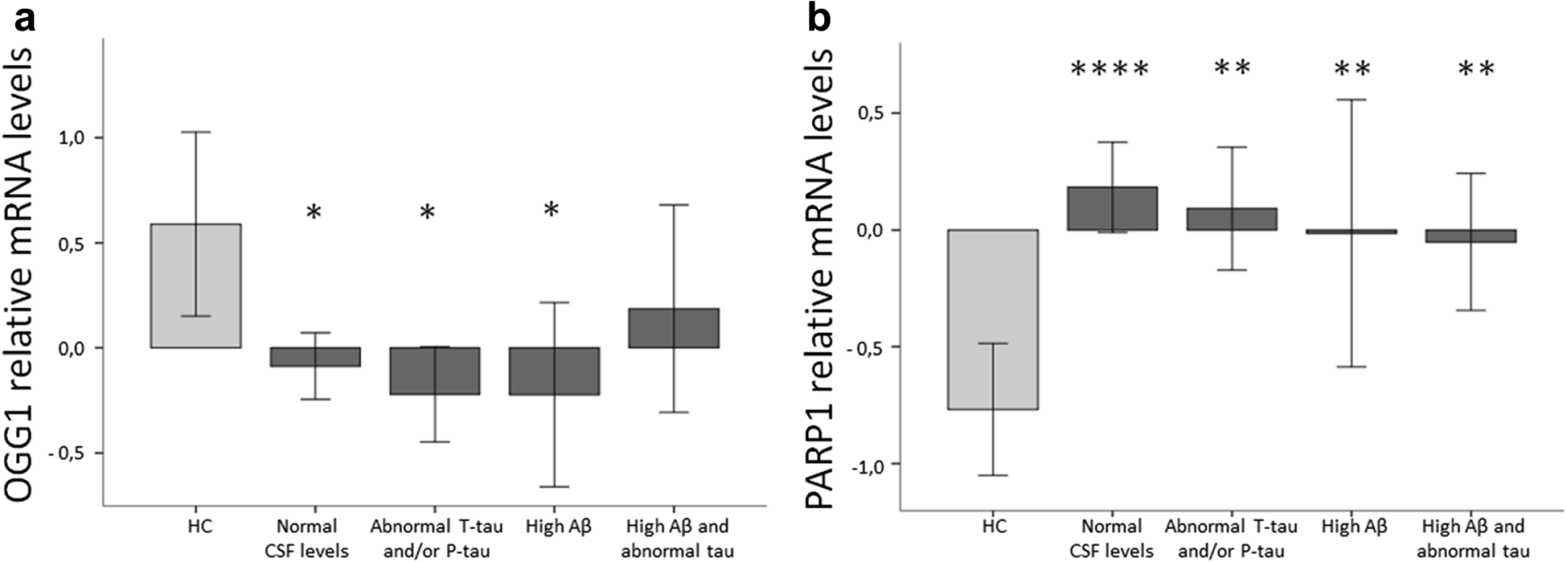
Relative mRNA levels of *OGG1* and PARP1 in CSF biomarker groups (mean ± SEM) in Aβ-42 and T-tau and/or P-tau, Aβ-42, T-tau and/or P-tau, patients with normal CSF levels of Aβ-42 and tau and healthy controls (HC). *p-values*: * < 0.05, ** < 0.01, *** < 0.001, **** < 0.0001. **a** The box plot show that mRNA levels of *OGG1* are significantly reduced in the groups: patients with normal CSF levels of Aβ-42 and tau (*p <0.05*), abnormal tau (*p <0.05*) and high Aβ-42 levels (*p <0.05*) compared to HC. **b** The box plot show that mRNA levels of *PARP1* are significantly increased in the groups: patients with normal CSF levels of Aβ-42 and tau (*p <0.0001*), abnormal tau levels (*p <0.01*) and high Aβ-42 levels (*p <0.01*) and both tau and Aβ-42 (*p <0.01*) compared to HC

The neuropathological hallmarks of AD are Aβ plaques and NFT and the biomarkers currently used to assess these features are the levels of Aβ-42 and tau in CSF. To explore the relations between BER and the pathological features of AD, we compared the BER mRNA profile in blood in individuals exhibiting 1) only high Aβ-42 (*n* = 19), 2) only low P-tau or high T-tau or both (referred to as abnormal tau from here on) (*n* = 24), 3) both high Aβ-42 and abnormal tau (*n* = 29) levels in CSF exceeding cut-off levels (see Additional file 1: Table S3), and 4) patients with normal levels of CSF Aβ-42 and tau (*n* = 64) and 5) HC (*n* = 28) displaying normal CSF levels of CSF Aβ-42 and tau. Thus, we have grouped the individuals according to biological correlates based on their CSF levels of Aβ-42 and tau and not by clinical disease status. For information on which patients are presented in each biological group, see Additional file 1: Table S4. *OGG1* mRNA was lower, in the patients with only high Aβ-42 levels (*p <0.05*), only abnormal tau levels (*p <0.05*) and patients with normal CSF levels of Aβ-42 and tau (*p <0.05*) than in HC. There were no significant differences in *OGG1* mRNA between patients with both abnormal Aβ-42 and tau levels and HC (Fig. 4a and Table 3). *PARP1* mRNA was significantly higher in all patients groups than in HC: e.g. high Aβ-42 levels (*p <0.01*), abnormal tau levels (*p <0.01*) and combined high Aβ-42 and abnormal tau (*p <0.01*), as well as patients with normal CSF levels of Aβ-42 and tau (*p <0.0001*) (Fig. 4b and Table 3). The abundance of *APE1* and *Polβ* mRNA was not correlated with any of the CSF biomarkers.

### Blood mRNA levels of *PARP1* decreases while *Polβ* increases with age

Former studies suggest that DNA repair capacity is altered with age so we wanted to explore how the BER mRNA profile changed over time. *PARP1* mRNA was negatively correlated with age (*p <0.05*) with an average effect per year of −0.009 (Table 3), while *Polβ* mRNA was positively correlated (*p <0.01*) with age with an average effect per year of 0.010 (Table 3). *APE1* and *OGG1* mRNA levels were not correlated with age.

### Correlation of Polβ levels in RNA deep sequencing and RT-qPCR

When comparing results from RNA deep sequencing and RT-qPCR there was a strong correlation between the expression of Polβ in both methods (95.7 % (*p = 0.00000009*), with particularly high expression of Polβ in the cerebellum samples. There was no correlation for APE1 (6.3 % (*p =0.83*), OGG1 (−1.8 % (*p = 0.95*) or PARP1 (−20.6 % (*p =0.48*).

### PARP1, APE1 and Polβ protein levels are modified in AD

**Fig. 5:**
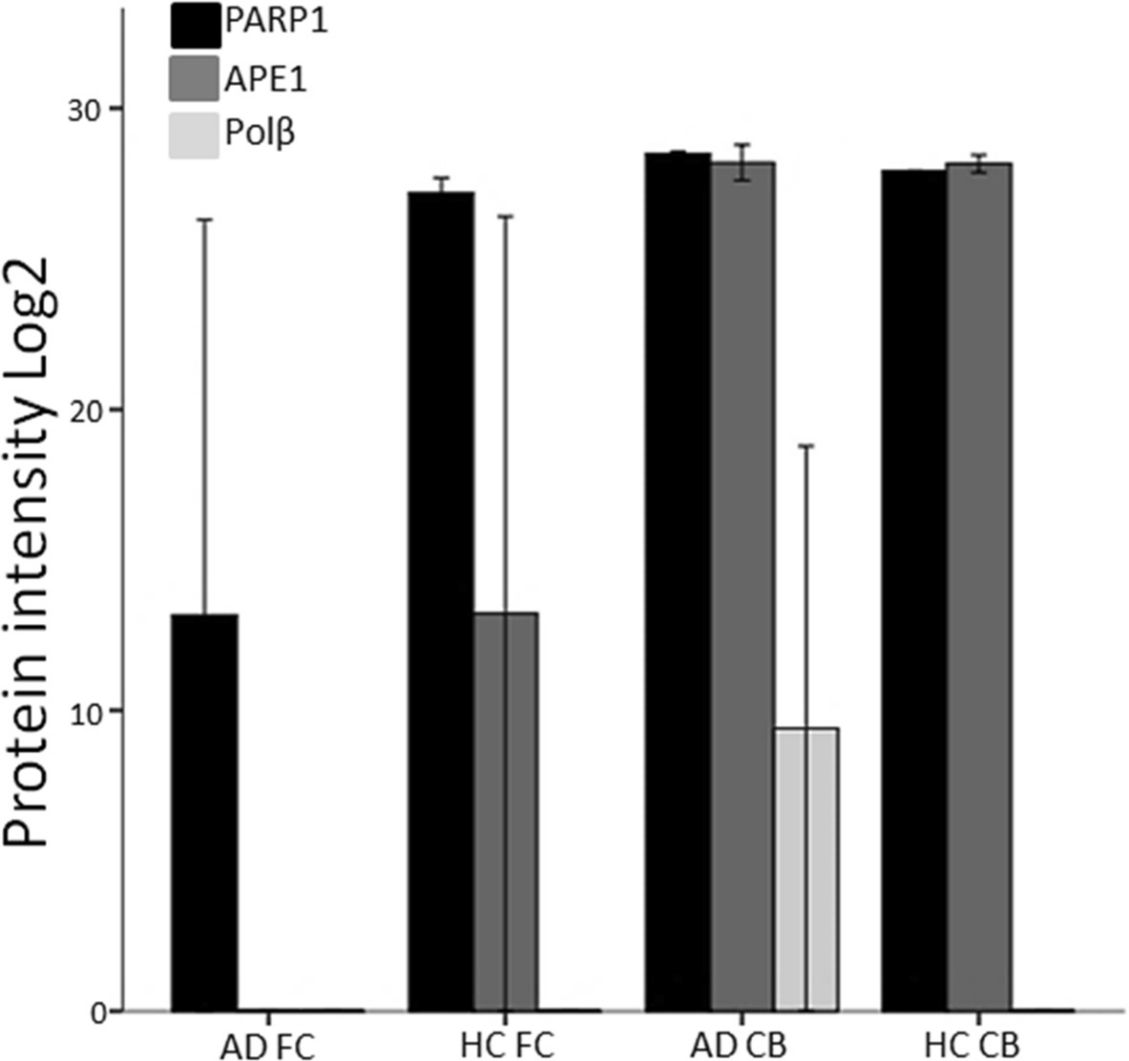
PARP1, APE1 and Polβ protein levels are modified in AD. Quantitative protein detection was performed by mass spectrometry (nLC and Thermo Q Exactive). Levels of base excision repair proteins PARP1, APE1 and Polβ were compared in Alzheimer’s patients (AD) and healthy controls (HC). Reference to house-keeping proteins is shown in Additional file 1: Figure S1. Black bars: PARP1, grey bars: APE1, light grey bars: Polβ. DNA glycosylase OGG1 was below the detection limit. Abbreviations: FC = Frontal cortex, CB = Cerebellum

Protein detection by next-generation mass spectrometry (MS) demonstrated relatively high protein levels of APE1 and PARP1 in the cerebellum of both AD and HC, while Polβ was only detected in AD cerebellum, however, at a lower level than APE1 and PARP1 (Fig. 5 and Additional file 1: Figure S1). APE1 was reduced in the frontal cortex of HC and totally absent in frontal cortex of AD patients, indicating a reduction of APE1 protein levels in the frontal cortex in general that is more pronounced in AD. PARP1 remained high in the frontal cortex of HC, but was reduced in the AD frontal cortex. OGG1 was not detected by MS in any of the samples.

## Discussion

The goal of this study was to investigate the levels of mRNAs and protein profiles of selected BER enzymes in brain tissue and blood, potentially as early markers of AD development. Notably, mRNA levels of *APE1, OGG1, Polβ* and *PARP1* were higher in brain tissue than in blood, reflecting the high energy consumption of the brain. Brain cells have a high metabolic rate with high glucose and oxygen turnover, creating substantial amounts of ROS. This oxidative stress, in combination with the post-mitotic state of neurons and a possible decreased ratio of antioxidant to pro-oxidant enzymes [32], make brain cells prone to be dependent on efficient and active BER repair. Our findings support the importance of BER in DNA repair in the brain, consistent with its high load of oxidative stress and DNA damage.

Notably, *Polβ* mRNA and protein levels were particularly high in the AD cerebellum compared to all other brain regions. This was also verified by RNA deep sequencing analysis, showing a 95,7 % correlation between the two transcriptomic methods (Additional file 1: Tables S7 and S8). The high level of Polβ may be a sign of late AD pathology, as the cerebellum remains free from tau-pathology until the most advanced Braak stage (VI), and neuronal loss and gliosis in this region has not been associated to early AD. Studies show that mice lacking Polβ have neonatal lethality with abnormal neurogenesis characterized by apoptotic cell death specifically only in the developing central and peripheral nervous system [33], implicating Polβ as an important factor for the nervous system already at the earliest stages of development. Neurons depend heavily on Polβ in the repair of oxidative DNA damage [34, 35], and single nucleotide gap-filling activity and protein level of Polβ was reduced in AD inferior parietal lobule (IPL) samples compared to HC [17]. Since it has been shown that other BER enzymatic activity, such as base excision, abasic site incision and nick ligation was not altered in brain tissue from AD patients, it has been suggested that Polβ is rate limiting for repair [17]. A study on the 3xTgAD/Polβ mouse model, displaying 50 % reduced Polβ activity, demonstrated significant increase in DNA damage accumulation. The reduced DNA repair capacity triggered neuronal death and hippocampal atrophy that did not occur in the 3xTgAD mouse. Thus, Polβ deficiency in combination with Aβ accumulation may comprise the ability of neurons to support synaptic activity to survive and render neurons vulnerability in reduction of cellular energy levels [36, 37], resulting in dysfunction and death. Growing evidence suggest a connection between AD and aspects of energy metabolism such as impairment in insulin [38] and insulin-like growth factor (IGF) signaling causing deficits in brain oxygen-glucose utilization and that the insulin resistance causes defects in the detoxification systems for oxidative stress [39]. PET imaging of AD brains demonstrate an AD related cerebral glucose metabolic covariance pattern with decreased metabolism in the temporoparietal regions and relatively increased metabolism in the subcortical white matter, cerebellum and sensorimotor cortex [40]. Thus, cerebellum presenting with high Polβ gene expression and increased metabolism may represent a compensatory mechanism for deficits in other brain regions or merely reflect a generally highly active brain region, potentially relating to the high density of granular cells in the cerebellar cortex [41]. Other studies have demonstrated lower levels of Polβ protein and Polβ activity in the cerebellum of AD patients compared to controls [17]. Our proteomic findings indicate that Polβ protein levels were lower than that of the other BER components and were only detectably increased in the AD cerebellum.

*APE1* mRNA was significantly lower in the entorhinal cortex of AD patients than in HC entorhinal cortex. The entorhinal cortex is one of the first regions to be affected in AD [42] and alterations observed here may represent early events of AD progression. However, the findings observed in post-mortem tissue represent late changes in the progression of AD. One study showed that APE1 proteins levels were similar in IPL and cerebellum tissue from AD and HC [17], while other studies demonstrated that APE1 protein expression was higher in brain tissue affected by AD pathology (hippocampus and surrounding temporal cortex) [43] and in cell extracts from AD patients [44]. Our proteomic data show relative high protein levels of APE1 in the cerebellum of both AD and HC, however, this was reduced in the frontal cortex of HC and totally absent in frontal cortex of AD patients, indicating a reduction of APE1 protein level in the frontal cortex in general that is accelerated in AD. APE1 has been shown to play a role in degrading damaged RNA [45], and oxidatively damaged RNA has been implicated as an important factor in neurodegeneration [46, 47].

In blood, *OGG1* mRNA transcript abundance was reduced in MCI, MCI/AD and AD patients compared to HC as well as in patients with abnormal levels of CSF Aβ-42 and tau and in patients with normal CSF levels of Aβ-42 and tau (mainly comprised of MCI and SCI patients). The results thus indicate that BER mRNA profile alterations occur independent of plaque and tau pathology in the progression of AD since the alterations are also seen in patients with no CSF pathology, but not in HC. This is consistent with findings from other studies [48]. OGG1 repairs oxidized guanine, and numerous studies show elevated oxidative lesions in both DNA and RNA in the prodromal phases of AD as well as in AD [18, 49–51]. It is suggested that oxidative DNA damage increases only during the early stages of AD and then decreases with the progression of the disease due to activation of a compensatory mechanism [52].

Since *OGG1* expression level was reduced in all groups except SCI, but also in patients with normal CSF levels of Aβ-42 and tau, we suggest that suppressed or reduced *OGG1* function is not directly associated with Aβ or tau pathology, but that lower *OGG1* transcript levels may indicate a DNA repair deficit in patients with MCI, MCI/AD and AD. Thus, OGG1 may represent a general marker for DNA repair deficits in subjects prone to develop AD [48]. However, it is important to emphasis that SCI and MCI are heterogeneous conditions that may or may not proceed to AD. Other studies show increased *OGG1* mRNA levels in brain tissue from the hippocampus, parahippocampal gyri and middle temporal gyri of patients with preclinical stages of AD compared to HC [18], suggesting that the elevation represents a compensatory increase in protein expression to moderate loss of activity due to posttranslational modification in response to increased oxidative DNA damage. MCI brain tissue did not exhibit a difference in OGG1 protein level compared to HC, but a significant decrease in OGG1 enzyme activity [53], proposing an association with increased post translational modification of OGG1 by 4-HNE. We were not able to detect OGG1 protein level by mass spectrometry in our study, suggesting low OGG1 levels in brain tissue from both HC and AD patient frontal cortex and cerebellum. Thus, OGG1 results from various studies are conflicting, and it still remains to be determined if *OGG1* mRNA and protein levels correspond to enzyme activity. OGG1 has also been implicated in immune system regulation and inflammation [54, 55], and it is suggested that there is a correlation between the efficiency of the DNA repair system and development of inflammation associated with the production of Th1 cytokines.

*PARP1* mRNA levels were higher in blood from all patient groups compared to HC. PARP1 is a general marker of DNA damage and inflammation and may also contribute to plaque formation and neurodegeneration in AD patients. Both plaque formation and neurodegeneration in the brain are associated with inflammation possibly mediated by NF-κB [56], a master regulator of the response to pro-inflammatory stimuli [56, 57] and cellular senescence [58]. PARP1 is required for NF-κB-dependent gene transcription [59] and NF-κB-dependent gene expression is associated with aging in mouse and humans [60]. Thus, PARP1 connects inflammation and the DNA damage response (DDR), through which excessive DNA damage can lead to cellular senescence. Senescent cells secrete pro-inflammatory cytokines, feeding a vicious cycle [56].

*PARP1* mRNA levels were also higher in patients with high levels of CSF Aβ-42 and abnormal tau and the two combined. However, *PARP1* mRNA levels were also higher in patients with normal CSF levels (comprised mainly by MCI and SCI patients) than in HC, indicating that there are processes relevant for AD development (inflammation, senescence, apoptosis and possibly also DNA repair deficiency) that are independent of tau and Aβ pathology. Thus, PARP1 may be a general early indicator of these other processes in all these groups as these changes are already evident in the SCI stage. In AD frontal cortex tissue, however, PARP1 protein levels were lower than in HC, reflecting late stage disease. One study demonstrated that PARP1 activation causes neuronal death in the hippocampal CA1 region by increasing the expression of Ca^2+^-permeable AMPA receptors [61], suggesting that increased PARP1 may cause damage to neurons. PARP1 thus functions at the center of cellular stress responses, where it processes diverse signals and, in response, directs cells to specific fates based on the type and strength of the stress stimulus. Thus, PARP1-stimulated senescence, apoptosis or necrosis could further deplete the pool of regenerative cells, and thereby contribute to neurodegeneration [62].

Consistent with other studies, we found that *PARP1* expression in blood decreased with age [63], while *Polβ* increased with age. Total BER capacity has been shown to be inversely correlated with age in healthy controls, but not in AD patients, however, reduced BER associated with AD regardless of age has been suggested to be linked to a premature ageing phenotype [17].

DNA repair capacity and protein levels differs among ethnic groups and there is considerable inter-individual variation [64]. Some of this enzymatic variation is most likely to be due to post-translational modifications. Our data demonstrated that the integrity of mRNA in post-mortem brain tissue was intact, as false negative results could have been an issue if mRNA levels were lower in brain due to post-mortem degradation. The transcriptomic analysis was reproducible and standardized equally for the two clinical cohorts, controlling for tissue differences and normalized in multiple steps to assure correct analysis, including the validation of GAPDH. Even though cohort differences might explain some of the differences in gene expression observed between blood and brain, the considerably higher level of gene expression measured in the brain than in blood is beyond inconsistent findings resulting from individual variability or from bias in the cohort of post-mortem samples. The discrepancy between transcriptomic and proteomic findings are most likely due to post-transcriptional processes, where high gene transcription levels not always reflected in a similar protein level.

## Conclusions

In summary, the data presented here provide novel insight into the early pathophysiology of AD, and OGG1 and PARP1 can potentially contribute as part of a set of blood biomarkers for identifying incipient AD. Early prediction of insipient AD or AD-like pathology is critical to the management of this disease and may also facilitate the design and evaluation of diagnostic, preventive and therapeutic tools for AD and AD-like forms of dementia.

## Abbreviations

AD, Alzheimer’s disease; APE1, AP endonuclease 1; Aβ, Amyloid abeta; BER, Base excision repair; CSF, Cerebrospinal fluid; HC, Healthy controls; MCI, Mild cognitive impairment; NFTs, Neurofibrillary tangles; OGG1, 8-Oxoguanine glycosylase; PARP1, Poly [ADP-ribose] polymerase 1; Polβ, Polymerase β; P-tau, Phosphorylated tau; ROS, Reactive oxygen species SCI, Subjective cognitive impairment; T-tau, Total tau. 

## Additional file

Additional file 1: Supplementary material. Altered DNA base excision repair profile in brain tissue and blood in Alzheimer’s disease. (DOCX 142 kb)
