# Supplementary material for: Altered DNA base excision repair profile in brain tissue and blood in Alzheimer’s disease
Source: Mol Brain. 2016 May 28;9:61. doi: 10.1186/s13041-016-0237-z (PMC4884418; doi:10.1186/s13041-016-0237-z)
Supplement: Supplementary file 1 — Supplementary material. Altered DNA base excision repair profile in brain tissue and blood in Alzheimer’s disease. (DOCX 142 kb) [file 13041_2016_237_MOESM1_ESM.docx]

**Supplementary material**

**Altered DNA base excision repair profile in brain tissue and blood in Alzheimer’s disease**

Meryl S. Lillenes^1,2^, Alberto Rabano^3^, Mari Støen^1,2^, Tahira Riaz^1^, Dorna Misaghian^1,2^, Linda Møllersen^1^, Ying Esbensen^4^, Clara-Cecilie Günther^5^, Per Selnes^6^, Vidar T. V. Stenset^6^, Tormod Fladby^6^ and Tone Tønjum^1,2^

^1^ Department of Microbiology, Oslo University Hospital, Norway

^2^ Department of Microbiology, University of Oslo, Norway

^3^ Fundación Centro Investigación Enfermedades Neurológicas (CIEN), Spain

^4^ Department of Clinical Molecular Biology and Laboratory Sciences (EpiGen), Division of Medicine, Akershus University Hospital and University of Oslo, Norway

^5^ Norwegian Computing Center, Norway

^6^ Department of Neurology, Faculty Division, Akershus University Hospital, University of Oslo, Norway

*Correspondence: Tone Tønjum, Healthy Brain Aging Centre (HBAC), Department of Microbiology, University of Oslo, Oslo University Hospital, Postbox 4950 Nydalen, NO-0424 Oslo, Norway. Tel. +47 23079018; *Email address*: [tone.tonjum@medisin.uio.no](mailto:tone.tonjum@medisin.uio.no)

Co-authors email addresses: Meryl S. Lillenes: [m.s.lillenes@medisin.uio.no](mailto:m.s.lillenes@medisin.uio.no); Alberto Rabano: arabano@fundacioncien.es; Mari Støen: [Mari.Stoen@rr-research.no](mailto:Mari.Stoen@rr-research.no); Tahira Riaz: [tahira.riaz@medisin.uio.no](mailto:tahira.riaz@medisin.uio.no); Dorna Misaghian: [dorna.misaghiyan@gmail.com](mailto:dorna.misaghiyan@gmail.com); Linda Møllersen: [linda.mollersen@gmail.com](mailto:linda.mollersen@gmail.com); Ying Esbensen: [q.y.esbensen@medisin.uio.no](mailto:q.y.esbensen@medisin.uio.no); Clara-Cecilie Günther: [clara-cecilie.gunther@nr.no](mailto:clara-cecilie.gunther@nr.no); Per Selnes: [per.selnes@medisin.uio.no](mailto:per.selnes@medisin.uio.no); Vidar T. V. Stenset: [uxstvh@ous-hf.no](mailto:uxstvh@ous-hf.no); Tormod Fladby: [tormod.fladby@medisin.uio.no](mailto:tormod.fladby@medisin.uio.no)

Key words: Alzheimer’s disease; oxidative DNA damage, DNA repair, base excision repair, ; gene expression; DNA glycosylase hOGG1; PARP1; APE1; Polβ, human brain biopsies

Running title: DNA repair in Alzheimer’s disease

**Table S1 Clinical characteristics of the blood sample patient cohort**

| **Clinical parameter**  **Mean (±SD)** | **HC**  **Total n = 28** | | **SCI**  **Total n = 24** | | **MCI**  **Total n = 45** | | **MCI/AD**  **Total n = 28** | | **AD dementia**  **Total n = 41** | |
| --- | --- | --- | --- | --- | --- | --- | --- | --- | --- | --- |
| **Age (range)** | 65.1(49-80) | 28 | 62.3 (50-77) | 24 | 66.1 (52-90) | 45 | 69.6 (53-81) | 28 | 70.6 (51-84) | 41 |
| **Men/women** | 14/14 | 28 | 15/9 | 24 | 27/18 | 45 | 12/16 | 28 | 19/22 | 41 |
| **MMSE** | - | 0 | 29 (1.77) | 17 | 27.5 (2.2) | 43 | 26.9 (1.98) | 28 | 18.5 (5.05) | 36 |
| **CSF T-tau (ng/L)** | 305 (157.2) | 27 | 266 (114.5) | 24 | 315 (140.1) | 44 | 479 (276.8) | 28 | 769 (396,9) | 40 |
| **CSF P-tau (ng/L)** | 47 (17.7) | 27 | 47 (14.4) | 24 | 56 (15.9) | 44 | 83 (41.5) | 28 | 93 (42.0) | 40 |
| **CSF Aβ-42 (ng/L)** | 932 (226) | 27 | 949 (224.5) | 24 | 888 (250.6) | 44 | 515 (213.9) | 28 | 548 (211.1) | 38 |

Abbreviations: SCI = subjective cognitive impairment, MCI = mild cognitive impairment, MCI/AD = mild cognitive impairment due to Alzheimer’s disease, AD dementia = Alzheimer’s disease with clinical dementia, MMSE = Mini Mental State Examination, CSF = cerebral spinal fluid, T-tau = total tau, P-tau = phosphorylated tau, GE = gene expression.

**Table S2 Clinical characteristics of the brain tissue patient cohort**

| **Subjects** | **Age** | **Gender** | **PMI** | **NP** | **BS** | **MMSE** | **APOE** | **Cause of death** |
| --- | --- | --- | --- | --- | --- | --- | --- | --- |
| **Alzheimer’s patients** | | | | | | | | |
| 1 | 57 | Female | 5 | 3 | VI | NA | NA | Respiratory infection |
| 2 | 66 | Female | 5 | 3 | VI | NA | NA | NA |
| 3 | 73 | Female | 11 | 3 | V | 0 | e4/e4 | Acute myocardial infarction |
| 4 | 77 | Female | 6 | 3 | VI | 12 | e3/34 | Sepsis |
| 5 | 79 | Female | 4 | 3 | V | NA | NA | Respiratory infection |
| 6 | 79 | Female | 3 | 3 | VI | NA | NA | NA |
| 7 | 82 | Female | 4.5 | 3 | V | 0 | e3/e4 | Lower intestinal haemorrhage |
| 8 | 83 | Female | 5 | 3 | V | 0 | e3/e4 | NA |
| 9 | 84 | Female | 2 | 3 | V | 1 | e3/e4 | NA |
| 10 | 84 | Female | 5.5 | 3 | VI | NA | NA | NA |
| 11 | 86 | Female | 6 | 3 | VI | NA | NA | Respiratory failure |
| 12 | 86 | Female | 7 | 3 | V | NA | NA | NA |
| 13 | 86 | Female | 5 | 3 | V | NA | NA | NA |
| 14 | 87 | Female | 5 | 3 | VI | NA | NA | Cachexia |
| 15 | 88 | Female | 5 | 3 | VI | NA | NA | NA |
| 16 | 89 | Female | 4.5 | 3 | V | NA | NA | NA |
| 17 | 90 | Female | 7 | 3 | V | 2 | e3/e3 | Pulmonary thromboembolism |
| 18 | 91 | Female | 5 | 3 | V | NA | NA | Cardiac |
| 19 | 91 | Female | 3 | 3 | VI | 0 | e3/e4 | NA |
| 20 | 92 | Female | 1.5 | 3 | VI | 2 | e3/e3 | NA |
| 21 | 94 | Female | 4 | 3 | V | 0 | e3/e3 | Multiorgan failure |
| 22 | 98 | Female | 4 | 2 | IV | NA | NA | NA |
| 23 | 66 | Male | 5 | 3 | V | NA | NA | Respiratory infection |
| 24 | 68 | Male | 10 | 3 | VI | NA | NA | Respiratory failure. |
| 25 | 71 | Male | 6 | 3 | V | NA | NA | Sepsis |
| 26 | 73 | Male | 5 | 3 | V | NA | NA | NA |
| 27 | 75 | Male | 5 | 3 | V | NA | NA | Sepsis, neutropenia |
| 28 | 79 | Male | 4 | 3 | VI | NA | e3/e4 | Respiratory infection |
| 29 | 80 | Male | 5 | 3 | IV | 14 | e3/e4 | Respiratory infection |
| 30 | 80 | Male | 5 | 3 | V | NA | NA | NA |
| 31 | 80 | Male | 6 | 3 | V | NA | NA | NA |
| 32 | 81 | Male | 2.5 | 3 | V | NA | NA | Cardiac failure |
| 33 | 82 | Male | 2 | 3 | V | NA | NA | Respiratory infection |
| 34 | 83 | Male | 9 | 3 | VI | NA | NA | Respiratory infection + PTE |
| 35 | 84 | Male | 4 | 3 | VI | NA | NA | NA |
| 36 | 85 | Male | 2.5 | 3 | VI | 1 | e3/e4 | NA |
| 37 | 86 | Male | 4 | 2 | V | 14 | NA | Respiratory failure |
| 38 | 87 | Male | 6 | 3 | V | NA | NA | Sepsis |
| 39 | 87 | Male | 10 | 3 | VI | NA | NA | NA |
| 40 | 88 | Male | NA | 2 | IV | NA | NA | NA |
| 41 | 91 | Male | 6 | 3 | V | NA | NA | NA |
| 42 | 92 | Male | 6 | 3 | VI | 12 | e3/e4 | NA |
| **Controls** | | | | | | | | |
| 1 | 52 | Female | 6 | 0 | 0 | NA | NA | Breast cancer |
| 2 | 58 | Female | 3 | 0 | 0 | NA | NA | NA |
| 3 | 59 | Female | 2 | 0 | 0 | NA | NA | Acute pancreatitis |
| 4 | 62 | Female | 2 | 0 | 0 | NA | NA | Sepsis |
| 5 | 46 | Male | 3 | 0 | 0 | NA | NA | Trauma (Forensic) |
| 6 | 54 | Male | 6 | 0 | 0 | NA | NA | Acute myocardial infarction |
| 7 | 63 | Male | 2 | 0 | I | NA | NA | Pancreas cancer |
| 8 | 77 | Male | 5 | 0 | I | NA | NA | Lung cancer |
| 9 | 84 | Male | 3 | 0 | I | NA | NA | Cardiomyopathy |

**Table S3 Reference values of clinical analyses**

| **Analysis** | **Cut-off values** |
| --- | --- |
| CSF T-tau: | ≥ 300 ng/L for ages under 50 years |
|  | ≥450 ng/L for ages 50-69 years and |
|  | ≥ 500 ng/L for ages 70 and over. |
| CSF P-Tau: | < 80 ng/L |
| CSF Aβ42: | < 550 ng/L |

CSF = cerebral spinal fluid, T-tau = total tau, P-tau = phosphorylated tau.

**Table S4 Distribution of the different patient groups in the biological cerebral spinal fluid categories**

| **Cerebral spinal fluid biomarkers** | **HC** | **SCI** | **MCI** | **MCI/AD** | **AD dementia** | **Total** |
| --- | --- | --- | --- | --- | --- | --- |
| HC (normal CSF levels) | 28 | 0 | 0 | 0 | 0 | **28** |
| Patients with normal CSF levels | 0 | 22 | 41 | 0 | 1 | **65** |
| Patients with high t-tau and/or low p-tau levels | 0 | 2 | 1 | 6 | 15 | **24** |
| Patients with high Aβ levels | 0 | 0 | 1 | 12 | 6 | **19** |
| Patients with both abnormal Aβ and Tau levels | 0 | 0 | 1 | 10 | 18 | **29** |
| **Total** | **28** | **24** | **44** | **28** | **40** | **164** |

Abbreviations: CSF = cerebral spinal fluid, HC = healthy controls, SCI = subjective cognitive impairment, MCI = mild cognitive impairment, MCI/AD = mild cognitive impairment due to Alzheimer’s disease, AD dementia = Alzheimer’s disease with clinical dementia. CSF data are missing on 1 MCI and 1 AD dementia patients.

**Table S5 APOE frequencies in the different patient groups in the cohort**

| **APOE alleles** | **HC** | **SCI** | **MCI** | **MCI/AD** | **AD dementia** | **Total** |
| --- | --- | --- | --- | --- | --- | --- |
| **2/2** | 0 | 0 | 0 | 0 | 1 | **1** |
| **2/3** | 5 | 6 | 1 | 1 | 0 | **13** |
| **2/4** | 0 | 1 | 0 | 0 | 2 | **3** |
| **3/3** | 17 | 12 | 26 | 7 | 11 | **73** |
| **¾** | 5 | 4 | 14 | 12 | 17 | **52** |
| **4/4** | 1 | 0 | 4 | 8 | 10 | **23** |
| **Total** | **28** | **23** | **45** | **28** | **41** | **165** |

Abbreviations: Healthy controls = HC, APOE data is missing on one SCI patient.

**Table S6 TaqMan qRT-PCR gene expression assay specifics**

| **Gene** | **ABI assay ID** | **Part number** | **Interrogated Sequence RefSeq** | **Translated Protein**  **RefSeq** | **Dye label** | **NCBI Location Chromosome** | **Exon Boundary** |
| --- | --- | --- | --- | --- | --- | --- | --- |
| APE1 | Hs00172396_m1 | 4351370 | NM_001244249.1 | NP_001231178.1 | FAM | Chr.14: 20923290 - 20925926 | 3-4 |
| OGG1 | Hs00213454_m1 | 4351370 | NM_002542.5 | NP_002533.1 | FAM | Chr.3: 9791628 - 9808353 | 2-3 |
| Polβ | Hs01099715_m1 | 4351370 | NM_002690.2 | NP_002681.1 | FAM | Chr.8: 42196030 - 42229313 | 13-14 |
| PARP1 | Hs00242302_m1 | 4351370 | NM_001618.3 | NP_001609.2 | FAM | Chr.1: 226548392 - 226595801 | 7-8 |
| GAPDH | Hs99999905_m1 | 4351370 | NM_002046.4 | NP_002037.2 | FAM | Chr.12: 6643657 – 6647536 | 3-3 |

**Table S7 Relative mRNA levels of APE1, OGG1, Polβ and PARP1 in blood and brain tissue measured by qRT-PCR**

|  | | | **Relative mRNA levels (mean, SD, range)** | | | |
| --- | --- | --- | --- | --- | --- | --- |
| **Patient group** | **Material (n)** | **Brain part (n)** | **APE1** | **OGG1** | **Polβ** | **PARP1** |
| **Healthy controls** | Blood (28) |  | 1.02 SD 0.2  (0.60-1.40) | 1.02 SD 0.23  (0.69-1.46) | 0.78 SD 0.2  (0.41-1.11) | 0.87 SD 0.32  (0.51-1.84) |
|  | Brain tissue (9) | All (33) | 1.76 SD 0.89  (0.60-5.46) | 3.27 SD 2.49  (0.74-11.07) | 6.2 SD 9.29  (0.64-41.59) | 2.85 SD 2.72  (0.68-16.09) |
|  |  | FC (9) | 1.41 SD 0.40  (0.81-2.19) | 2.69 SD 1.99  (1.25-7.16) | 1.36 SD 0.68  (0.79-2.60) | 1.83 SD 1.21  (0.68-4.47) |
|  |  | EC (8) | 2.36 SD 1.46  (0.60-5.46) | 3.01 SD 3.32  (0.74-11.07) | 1.2 SD 0.37  (0.64-1.61) | 2.15 SD1.09  (0.84-3.85) |
|  |  | HC (7) | 1.16 SD 0.19  (0.79-1.33) | 1.85 SD 0.93  (1.08-3.43) | 1.36 SD 0.33  (0.91-1.77) | 1.81 SD 0.60  (1.01-2.78) |
|  |  | CB (9) | 2.03 SD 0.48  (1.61-2.93) | 5.18 SD 2.05  (3.01-9.12) | 18.69 SD 9.49  (9.04-41.59) | 5.31 SD 4.16  (2.83-16.09) |
| **SCI** | Blood (24) |  | 1.09 SD 0.41  (0.55-2.54) | 0.94 SD 0.18  (0.62-1.28) | 0.90 SD 0.63  (0.33-3.30) | 1.32 SD 0.47  (0.63-2.88) |
| **MCI** | Blood (45) |  | 0.96 SD 0.29  (0.44-1.60) | 0.88 SD 0.18  (0.49-1.27) | 0.82 SD 0.37  (0.23-1.8) | 1.23 SD 0.38  (0.55-2.24) |
| **MCI/AD** | Blood (28) |  | 0.96 SD 0.29  (0.48-2.04) | 0.90 SD 0.21  (0.58-1.61) | 0.89 SD 0.32  (0.41-1.65) | 1.34 SD 0.57  (0.53-3.40) |
| **AD dementia** | Blood (41) |  | 1.06 SD 0.34  (0.61-2.32) | 0.90 SD 0.27  (0.59-2.37) | 0.99 SD 0.43  (0.47-2.10) | 1.14 SD 0.32  (0.22-1.71) |
|  | Brain tissue (42) | All (154) | 1.73 SD 0.68  (0.56-4.23) | 4.38 SD 2.55  (0.78-19.60) | 8.31 SD 12.55  (0.53-54.09) | 3.45 SD 1.98  (0.78-9.64) |
|  |  | FC (37) | 1.55 SD 0.63  (0.56-3.21) | 3.23 SD 1.46  (1.05-5.98) | 1.75 SD 1.01  (0.53-4.87) | 2.17 SD 0.89  (0.78-4.20) |
|  |  | EC (39) | 1.73 SD 0.59  (0.72-3.14) | 3.93 SD 1.69  (1.17-7.53) | 2.37 SD 0.95  (0.89-4.47) | 4.87 SD 2.36  (1.26-9.64) |
|  |  | HC (39) | 1.47 SD 0.47  (0.71-2.65) | 3.91 SD 2.09  (0.78-9.47) | 1.59 SD 0.75  (0.61-3.87) | 3.87 SD 1.93  (1.05-9.01) |
|  |  | CB (36) | 2.19 SD 0.80  (0.97-4.23) | 6.51 SD 3.43  (3.00-19.60) | 28.62 SD 11.59  (4.89-54.09) | 2.86 SD 1.24  (0.95-5.65) |

Abbreviations: FC = Frontal cortex, EC = Entorhinal cortex, HC = Hippocampus, CB = Cerebellum. Analysis of brain tissue from the 42 AD patients are based on 154 valid samples, whereas 14 single samples were excluded due to poor quality. However, all 42 patients are included as viable samples were obtained from some brain regions and not all.

**Table S8 Transcriptomic / RNA deep sequencing results (Fragments per kilobase Million (FPKM)) of APE1, OGG1, Polβ and PARP1 mRNAs in human brain tissue**

| Brain part | AD | APE1 | OGG1 | Polβ | PARP1 |
| --- | --- | --- | --- | --- | --- |
| Frontal cortex | AD | 37.4 | 9.7 | 11.4 | 24.9 |
| Frontal cortex | AD | 41.6 | 7.2 | 10.2 | 33.2 |
| Frontal cortex | HC | 41.8 | 6.5 | 14.7 | 27.5 |
| Frontal cortex | HC | 35.8 | 6.4 | 12 | 40.5 |
|  |  |  |  |  |  |
| Cerebellum | AD | 31.1 | 5.9 | 73 | 38.2 |
| Cerebellum | AD | 36.7 | 7.6 | 88.3 | 30.2 |
| Cerebellum | HC | 34.7 | 10.6 | 96.7 | 26.4 |
| Cerebellum | HC | 33.9 | 8.9 | 56.5 | 28.2 |
|  |  |  |  |  |  |
| Hippocampus | AD | 34.6 | 10.2 | 9.7 | 40.1 |
| Hippocampus | AD | 41.4 | 13.1 | 9.7 | 39.5 |
| Hippocampus | HC | 52.8 | 9.2 | 8.4 | 29.3 |
| Hippocampus | HC | 34.8 | 11.8 | 7 | 30.1 |
|  |  |  |  |  |  |
| Entorhinal cortex | AD | 46.1 | 6.5 | 10.1 | 38.9 |
| Entorhinal cortex | HC | 48.7 | 10.4 | 11.4 | 36.7 |

Abbreviations: AD = Alzheimer’s disease, HC = healthy controls

**Table S9. Protein detection by next-generation mass spectrometry (MS)**

| **Gene name** | | | **APE1** | **APOE** | **APP** | **AQP4** | **GAPDH** | **PARP1** | **POLB** | **RAD23B** |
| --- | --- | --- | --- | --- | --- | --- | --- | --- | --- | --- |
| **Sequence coverage [%]** | | | 51.6 | 78.5 | 14.7 | 26.3 | 90.4 | 26.7 | 5.7 | 38.4 |
| **Peptides** | | | 12 | 25 | 9 | 9 | 25 | 19 | 2 | 15 |
| **Unique peptides** | | | 12 | 20 | 9 | 9 | 3 | 19 | 2 | 12 |
| **Brain part** | | **MS values** |  |  |  |  |  |  |  |  |
|  |  | **LFQ intensity** | NaN | 2.7E+08 | NaN | 1.1E+08 | 5.9E+09 | 8.4E+07 | NaN | 6.4E+07 |
|  | **FC 266** | **Peptides** | 1 | 14 | 2 | 8 | 22 | 3 | 0 | 7 |
| **AD** |  | **Unique peptides** | 1 | 11 | 2 | 8 | 3 | 3 | 0 | 4 |
|  |  | **LFQ intensity** | NaN | 1.1E+09 | NaN | 1.4E+08 | NaN | NaN | NaN | 7.5E+07 |
|  | **FC 166** | **Peptides** | 1 | 12 | 1 | 4 | 16 | 1 | 0 | 3 |
|  |  | **Unique peptides** | 1 | 8 | 1 | 3 | 1 | 1 | 0 | 3 |
|  |  | **LFQ intensity** | 2.1E+08 | 2.1E+08 | NaN | 1.3E+08 | 7E+09 | 4E+08 | 451060 | 7.4E+07 |
|  | **CB 266** | **Peptides** | 4 | 9 | 0 | 5 | 23 | 7 | 1 | 5 |
|  |  | **Unique peptides** | 4 | 6 | 0 | 5 | 3 | 7 | 1 | 5 |
|  |  | **LFQ intensity** | 4.6E+08 | 3.8E+08 | NaN | 3.4E+08 | 1.5E+08 | 3.7E+08 | NaN | 4.6E+08 |
|  | **CB 213** | **Peptides** | 6 | 13 | 1 | 5 | 21 | 9 | 1 | 8 |
|  |  | **Unique peptides** | 6 | 9 | 1 | 5 | 3 | 9 | 1 | 5 |
| **HC** |  | **LFQ intensity** | NaN | 1.6E+08 | NaN | 1.5E+07 | NaN | 2.2E+08 | NaN | 1.2E+08 |
|  | **FC 98** | **Peptides** | 1 | 4 | 0 | 2 | 15 | 2 | 0 | 4 |
|  |  | **Unique peptides** | 1 | 3 | 0 | 2 | 1 | 2 | 0 | 3 |
|  |  | **LFQ intensity** | 8.9E+07 | 2.6E+08 | NaN | 4.4E+08 | 3.5E+08 | 1.1E+08 | NaN | 1.9E+08 |
|  | **FC 177** | **Peptides** | 2 | 7 | 1 | 3 | 23 | 1 | 0 | 5 |
|  |  | **Unique peptides** | 2 | 6 | 1 | 3 | 3 | 1 | 0 | 2 |
|  |  | **LFQ intensity** | 2.5E+08 | 1.8E+08 | NaN | 3E+07 | 4.1E+09 | 2.5E+08 | NaN | 1.7E+08 |
|  | **CB 98** | **Peptides** | 7 | 15 | 0 | 4 | 22 | 8 | 1 | 6 |
|  |  | **Unique peptides** | 7 | 8 | 0 | 4 | 3 | 7 | 1 | 5 |
|  |  | **LFQ intensity** | 3.7E+08 | 9.8E+08 | NaN | 7.3E+08 | 1E+08 | 2.6E+08 | NaN | 8E+07 |
|  | **CB 177** | **Peptides** | 4 | 16 | 2 | 6 | 20 | 7 | 1 | 5 |
|  |  | **Unique peptides** | 4 | 11 | 2 | 6 | 3 | 7 | 1 | 5 |

Each protein identified by electrospray-based mass spectrometry is at the top of the table represented by its gene name and the collective total sequence coverage, number of peptides and unique peptides for that protein. In addition, for each sample, the quantitative numbers/LFQ intensity values, total peptides and unique peptides are provided separately. Abbreviations: AD = Alzheimer’s disease, HC = healthy controls, FC = frontal cortex, CB = cerebellum, NaN = not a number.

Protein intensity Log2


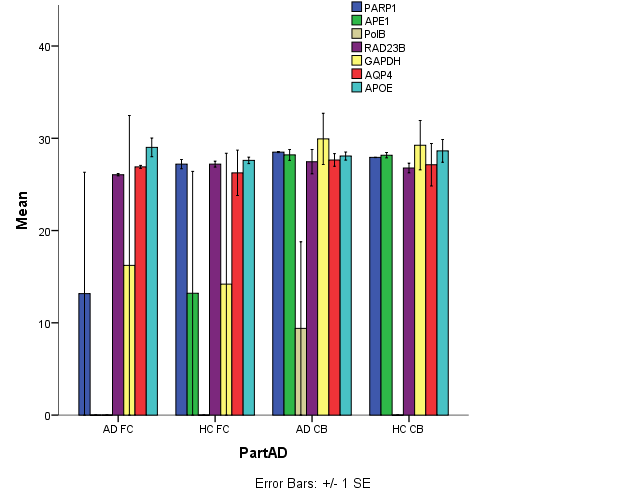


**Fig. S1 PARP1, APE1 and Polβ protein levels were altered in AD.** Protein levels of PARP1, APE1, Polβ, RAD23B, GAPDH, AQP4 and APOE in brain parts from AD patients and healthy controls (HC) (mean± 1 SEM). Quantitative protein detection was performed by mass spectrometry (nLC and Thermo Q Exactive). Relatively high protein levels of APE1 and PARP1 in the cerebellum (CB) of both AD and HC was detected, but APE1 was reduced in the frontal cortex (FC) of HC and totally absent in FC of AD patients, indicating a reduction of the APE1 protein level in the FC in general that is more pronounced in AD, while PARP1 remained high in the FC of HC but was reduced in AD FC. Polβ was detected only in AD CB, however, at a lower level than APE1 and PARP1. DNA glycosylase OGG1 was below the detection limit. RAD23B was detected in all samples in similar levels in all samples. GAPDH was detected in all CB and FC samples but was lower in FC compared to CB. AQP4 and APOE were detected in all samples and at similar levels in all samples. Blue bars: PARP1, green bars: APE1, light grey bars: Polβ, purple bars: RAD23B; yellow bars: GAPDH; red bars: Aquaporin 4, light blue bars: APOE. AD: Alzheimer’s disease patients (n = 2), HC: healthy controls (n = 2). FC: Frontal cortex, CB: Cerebellum.

**Methods and methodological considerations**

166 patients were recruited consecutively based on their referral to a university-hospital based memory clinic at Akershus university hospital in the time period 2000 and 2012.

*Diagnosis*

Distinct types of dementia are difficult to diagnose and classify, especially in early stages, partly due to the redundant symptomatology and frequently co-existing various etiologies. The diagnosis of AD is based on clinical and neuropsychological examination, the presence of cognitive impairment confirmed by neuropsychological testing along with the exclusion of other causes of dementia. The defined diagnosis of AD are outlined in the three publications [1-4], representing guidelines with the aim to secure AD diagnosis at early stages, prior to the onset of overt dementia. These documents represent a revision of the old criteria and include biomarkers of 1) Aβ accumulation, evident by abnormal tracer retention on amyloid positron emission tomography (PET) imaging and low CSF Aβ42, and 2) neuronal degeneration or injury, which are elevated CSF tau (both total-tau (T-tau), phosphorylated-tau (P-tau)), decreased fluorodeoxyglucose uptake on PET (FTG-PET) in a specific topographic pattern involving temporo-parietal cortex and atrophy on structural magnetic resonance imaging (MRI) with specific topographic patterns involving medial, basal and lateral temporal lobes and medial and lateral parietal cortices [1]. Patients with cognitive complaints are also classified using the Global deterioration scale (GDS) [5, 6], according to previously published procedures [7, 8] GDS 2 (SCI) patients achieved higher than a cutoff score, including score ≥28 for MMSE; GDS 3 patients (MCI) scored <28 on MMSE; GDS 4 or higher scored >0.5 on CDR or scored >1 for the sum of STEP variables 13–20; GDS 4 or higher individuals were considered demented. See the following reference for more on classification according to GDS score: [9-11]. Patients diagnosed with AD had a GDS score >3) and fulfilled additional criteria for AD [1-4]. The GDS scores for healthy controls were determined in a clinical interview. All normal controls used in this study were GDS 1.

However, diagnosis of definite AD can still only be made after post mortem histopathological confirmation [4, 12].

*Diagnostic eligibility criteria and patient groups*

AD, MCI and SCI are related conditions, and some clinical characteristics are shared among these three groups as well as subjectively healthy control patients. Clinical variation within each of these patient subgroups and the presence of variable comorbidities also complicate stratification of the cohort. However, we note 1) that the frequency of AD and non-AD dementia cases in this cohort is roughly similar to that in other cohorts [13], and 2) study participants were diagnosed by highly trained experts. Therefore, we argue 1) that the study presents the highest achievable accuracy with respect to stratification of a cohort of preclinical and demented AD patients and normal patients, and 2) we assert that we have provided reasonable measures for interpreting the data.

*Transcriptomic analysis and mRNA quantitation.*

*Isolation of RNA from blood and human brain biopsies.* Blood was collected in PAXgene RNA collection tubes (PreAnalytiX GmbH, Switzerland) and total RNA was extracted using the PAXgene Blood RNA kit (PreAnalytiX GmbH, Switzerland) designed specifically for RNA extraction from the PAX tubes. PAX tubes are considered the better choice to conserve RNA in blood as it contains preservatives that prevent RNA degradation, thus alternative collecting and RNA isolation methods used for tissue was not preferable in order to achieve the highest quality. As brain tissue is not optimally collected in PAX gene blood tubes, isolation using the PAXgene Blood RNA kit is inappropriate. Further, brain tissue contains much fat that makes RNA extraction particularly difficult. Several methods were tested in order to achieve appropriate quality measures and the best method for isolating brain tissue was PureLink® RNA Mini Kit. As both methods achieved high RIN values and satisfactory concentrations in general and the same amount of RNA for both materials were put into the cDNA synthesis using the same method (High cDNA reverse transcription kit, Applied Biosystems), we believe that the cDNA from the two methods are comparable. The major concern in this regard was more the potential degradation of RNA in brain tissue, but since mRNA levels appeared to be higher in brain than in blood, it shows that RNA is highly intact in fresh frozen brain tissue stored at -80 °C and our measures of quick handling time taking the frozen material out for RNA isolation and engage procedure ensured this quality. However, it is not to exclude that the mRNA levels could be even higher than measured despite of good RIN values, but we do not have false negative findings due to degraded RNA.

Isolation of RNA from fresh frozen post mortem human brain tissue was done manually and the samples were stored at -80℃ until total RNA was extracted. The samples were weighed and placed into MagNA Lyser Green Beads (Roche Diagnostics GmbH, Mannheim, Germany) with recommended amount of lysis buffer from PureLink® RNA Mini Kit (Ambion, Texas, USA) and then homogenized using MagNA Lyser instrument (Roche Diagnostics GmbH, Mannheim, Germany). The supernatant was collected, and total RNA was isolated manually using PureLink® RNA Mini Kit according to manufacturer’s recommendations and then eluted with RNAse-free water and RNAsecure™ RNase Inactivation Reagent (Ambion, Texas, USA) according to manufacturer’s recommendations for long term storage.

*Quantitative real time-PCR (qRT-PCR). Validation of reference genes.* The expression of 32 reference genes were validated using TagMan Human Endogenous Control Plates (Applied Biosystems, Foster City, CA, USA) and GAPDH was one of the most stable reference genes with the least variability across samples in blood from both AD patients and healthy controls (score 0.515, variation span among the 32 reference genes: 0.491-2.954). We did not choose the reference gene with the absolutely lowest score as that gene exhibited a high Ct value (>25 Ct) which is not recommended as it indicated larger variations between replicates, and GAPDH had a low score as well as an acceptable Ct value (< 25). Further, in order to compare blood and brain tissue, we also had to use the same reference gene, method and reagents for brain tissue. In addition to this, all experiments were analysed on the same StepOnePlus™ instrument using the same TaqMan Gene expression assays reagents and setup.

*qRT-PCR reactions.* Each qRT-PCR plate was set up for the standard curve method, using 5 dilutions for the standard curve for all genes. All standard curves were made from the same sample, thus also functioning as a positive control and reference sample between runs. A non-template control (NTC) was included for all genes in all plates. PCR efficiency was between 90-110 % for all standard curves for all genes at all plates, which gives standard curve slopes from – 3.6 to 3.1.

*Normalization of qRT-PCR data.* Normalization of data was performed at several levels. The same amount of blood was drawn in all PAX tubes, and the whole tube was used for isolation. Brain tissue was weighed, however, the amount of brain tissue available varied from each brain region: availability of hippocampus (HC) and entorhinal cortex (EC) was substantially more scarce than frontal cortex (FC) and cerebellum (CB). Thus, smaller amount was taken from these two brain parts. The average weight for RNA extraction from HC was 11.6mg (SD ±2,1mg), EC was 22.7 mg (SD ±4.7mg), FC was 39.1mg (SD ±5,5mg) and CB was 37.7mg (SD ±5,1mg). RNA concentrations of both blood and brain tissue was determined using ND-1000 spectrophotometer (NanoDrop technologies, Saveen & Werner AB, Sweden), however, this method do not reveal if RNA is degraded. Thus, RNA purity, integrity and yield were confirmed for all samples using Agilent 2001 Bioanalyzer and RNA 6000 Nano Kit (Agilent technologies, California, USA) according to the recommendations of the manufacturer. RNA samples with unsatisfactory purity (blood RIN<7, brain RIN<5) were isolated again until satisfactory purity and RIN value was obtained or dismissed. cDNA was prepared from 1000ng total RNA from blood and brain tissue in 100µL using the High cDNA reverse transcription kit (Applied Biosystems) according to the recommendations from the manufacturer (Invitrogen, US). Thus, normalizing to RNA quantity with reasonably precise quantification and quality assessment was performed, however, this does not control for differences in efficiency of the reverse transcription and real-time PCR reactions and pipetting errors. Normalizing to a reference gene reflects the variability if the reverse transcriptase transcribes or the DNA polymerase amplifies a target gene in two samples at different rates and represents the most thorough method of addressing most sources of variability. GAPDH expression is the most commonly used reference gene in qRT-PCR in blood and brain, however, the expression of this house-keeping gene has been shown to vary in different tissues/conditions etc [14]. The literature on GAPDH as a reference gene is conflicting where some studies also find GAPDH to be one of the most stable in human brain tissue from AD patients and controls and particularly in the cerebellum [15] as well as in human brain tissue and gliomas [16]. Other studies have simply excluded GAPDH from their reference gene validation due to poor efficiency of the primers (poor primer optimisation), thus not really addressing the stability of GAPDH as a reference gene in their material [17]. Yet, other studies have employed poor methods for monitoring the efficiency of gene expression analysis such as the Delta Delta Cq (ΔΔCt) method, which is the easiest, but also the oldest and least reliable method, assuming 100% efficiency for all runs which rarely occurs even with optimal primer pairs: e.g. falsely calculated differences in expression ratio of 47% in case of E target < E reference gene and 209% in case of E target> E ref after 25 performed cycles.

*Proteomics analyses by high-end mass spectrometry*

Peptide characterization and quantitation were performed by electrospray-based high resolution mass spectrometry (Q-Exactive, Thermo-Fischer). **Lysis.** Brain tissue was re-suspended in lysis solvent (4 times the volume of the pellet) containing 2% SDS/10 mM Tris-HCl, pH 7.5, supplemented with protease inhibitor cocktail (EDTA free, Roche) and PhosStop (Roche). The samples were transferred to Lysing Matrix B tubes and disrupted with MagNa Lyser instrument (Roche) in cycles until fully lysed. The lysates were centrifuged at 15.000x g for 15 minutes and the supernatant was collected. **In-solution digestion.** Prior to trypsin digestion, 100µg of protein lysates were precipitated over night with acetone at -20°C. The following day, the samples were centrifuged at 20.000xg and the supernatant was discarded. Air dried protein pellet was re-suspended in 10µl of 8M urea. Proteins were reduced with 1µl of 10mM DTT (Sigma-Aldrich) for 30 minutes, followed by alkylation with 1µl 50mM iodoacetamide (Sigma-Aldrich) for 30 minutes in the dark. The samples were then diluted with 40µl of 50mM ammonium bicarbonate and digested overnight with F1:100 of trypsin (sequencing grade modified, Promega, USA). **Sample fractionation.** The digested samples were fractionated by anion exchange column (Wisniewski JR et al. J. Proteome Res. 2009). **Mass spectrometry.** Samples were run on a Q-Exactive (Thermo Scientific, Germany) mass spectrometer coupled directly to an nLC (EASY 1000, Thermo Scientific, Germany) using a data-dependent Top10 method. A two-column set up was used with pre-column (Acclaim PepMap 100, 75µm x 2cm, nanoviper, C_18_, 3µm, 100Å, Thermo Scientific) and analytical column (PepMap RSLC, C18, 2µm, 100Å, 75µm x 50cm, Thermo Scientific). 6µl of sample was injected in triplicates. For peptide separation a 120 minutes gradient was used, with solvent A being 0.1% FA/3%ACN and solvent B being 0.1%FA/97% ACN (FA:LC-MS grade, Fluka; ACN: LC-MS grade, Merck). The mass spectrometer was operated at 70.000 resolution, AGC target at 1e6, maximum IT was sat to 200ms and the scan range was 300-1800*m/z* for MS scans. For MS/MS scans, the resolution was 17.500, AGC target 5e4, maximum IT at 100ms, isolation width was 2.0 *m/z* and NCE 28.0. The underfill ratio was set to 10.0% and dynamic exclusion at 30.0s. **Database search.** Mass spectrometry results were searched using MaxQuant software against the UniProt Knowledgebase (proteome ID: UP000005640). For a protein to be identified, minimum of two peptide identifications and one unique peptide hit was used as the threshold.

**References**

1. Jack CR, Jr., Albert MS, Knopman DS, McKhann GM, Sperling RA, Carrillo MC, Thies B, Phelps CH: **Introduction to the recommendations from the National Institute on Aging-Alzheimer's Association workgroups on diagnostic guidelines for Alzheimer's disease.** *Alzheimers Dement* 2011, **7:**257-262.

2. Sperling RA, Aisen PS, Beckett LA, Bennett DA, Craft S, Fagan AM, Iwatsubo T, Jack CR, Jr., Kaye J, Montine TJ, et al: **Toward defining the preclinical stages of Alzheimer's disease: recommendations from the National Institute on Aging-Alzheimer's Association workgroups on diagnostic guidelines for Alzheimer's disease.** *Alzheimers Dement* 2011, **7:**280-292.

3. Albert MS, DeKosky ST, Dickson D, Dubois B, Feldman HH, Fox NC, Gamst A, Holtzman DM, Jagust WJ, Petersen RC, et al: **The diagnosis of mild cognitive impairment due to Alzheimer's disease: recommendations from the National Institute on Aging-Alzheimer's Association workgroups on diagnostic guidelines for Alzheimer's disease.** *Alzheimers Dement* 2011, **7:**270-279.

4. McKhann GM, Knopman DS, Chertkow H, Hyman BT, Jack CR, Jr., Kawas CH, Klunk WE, Koroshetz WJ, Manly JJ, Mayeux R, et al: **The diagnosis of dementia due to Alzheimer's disease: recommendations from the National Institute on Aging-Alzheimer's Association workgroups on diagnostic guidelines for Alzheimer's disease.** *Alzheimers Dement* 2011, **7:**263-269.

5. Auer S, Reisberg B: **The GDS/FAST staging system.** *Int Psychogeriatr* 1997, **9 Suppl 1:**167-171.

6. Reisberg B, Ferris SH, de Leon MJ, Crook T: **Global Deterioration Scale (GDS).** *Psychopharmacol Bull* 1988, **24:**661-663.

7. Stenset V, Bjornerud A, Fjell AM, Walhovd KB, Hofoss D, Due-Tonnessen P, Gjerstad L, Fladby T: **Cingulum fiber diffusivity and CSF T-tau in patients with subjective and mild cognitive impairment.** *Neurobiol Aging* 2011, **32:**581-589.

8. Nordlund A, Rolstad S, Hellstrom P, Sjogren M, Hansen S, Wallin A: **The Goteborg MCI study: mild cognitive impairment is a heterogeneous condition.** *J Neurol Neurosurg Psychiatry* 2005, **76:**1485-1490.

9. Petersen RC, Jack CR, Jr.: **Imaging and biomarkers in early Alzheimer's disease and mild cognitive impairment.** *Clin Pharmacol Ther* 2009, **86:**438-441.

10. Petersen RC: **Mild cognitive impairment as a diagnostic entity.** *J Intern Med* 2004, **256:**183-194.

11. Reisberg B, Gauthier S: **Current evidence for subjective cognitive impairment (SCI) as the pre-mild cognitive impairment (MCI) stage of subsequently manifest Alzheimer's disease.** *Int Psychogeriatr* 2008, **20:**1-16.

12. McKhann G, Drachman D, Folstein M, Katzman R, Price D, Stadlan EM: **Clinical diagnosis of Alzheimer's disease: report of the NINCDS-ADRDA Work Group under the auspices of Department of Health and Human Services Task Force on Alzheimer's Disease.** *Neurology* 1984, **34:**939-944.

13. Shimada H, Ataka S, Takeuchi J, Mori H, Wada Y, Watanabe Y, Miki T: **Pittsburgh compound B-negative dementia: a possibility of misdiagnosis of patients with non-alzheimer disease-type dementia as having AD.** *J Geriatr Psychiatry Neurol* 2011, **24:**123-126.

14. Penna I, Vella S, Gigoni A, Russo C, Cancedda R, Pagano A: **Selection of candidate housekeeping genes for normalization in human postmortem brain samples.** *Int J Mol Sci* 2011, **12:**5461-5470.

15. Coulson DT, Brockbank S, Quinn JG, Murphy S, Ravid R, Irvine GB, Johnston JA: **Identification of valid reference genes for the normalization of RT qPCR gene expression data in human brain tissue.** *BMC Mol Biol* 2008, **9:**46.

16. Grube S, Gottig T, Freitag D, Ewald C, Kalff R, Walter J: **Selection of suitable reference genes for expression analysis in human glioma using RT-qPCR.** *J Neurooncol* 2015, **123:**35-42.

17. Wang Q, Ishikawa T, Michiue T, Zhu BL, Guan DW, Maeda H: **Stability of endogenous reference genes in postmortem human brains for normalization of quantitative real-time PCR data: comprehensive evaluation using geNorm, NormFinder, and BestKeeper.** *Int J Legal Med* 2012, **126:**943-952.
